# The role of ion solvation in lithium mediated nitrogen reduction†

**DOI:** 10.1039/d2ta07686a

**Published:** 2022-11-28

**Authors:** O. Westhead, M. Spry, A. Bagger, Z. Shen, H. Yadegari, S. Favero, R. Tort, M. Titirici, M. P. Ryan, R. Jervis, Y. Katayama, A. Aguadero, A. Regoutz, A. Grimaud, I. E. L. Stephens

**Affiliations:** a Department of Materials, Imperial College London UK i.stephens@imperial.ac.uk; b Solid-State Chemistry and Energy Laboratory, UMR8260, CNRS, Collège de France France alexis.grimaud@bc.edu; c Department of Chemistry, University of Copenhagen Denmark; d Department of Chemical Engineering, Imperial College London UK; e The Faraday Institution, Quad One, Harwell Science and Innovation Campus Didcot OX11 0RA UK; f Eletrochemical Innovation Lab, Department of Chemical Engineering, University College London UK; g SANKEN, Osaka University Japan; h Instituto de Ciencia de Materiales de Madrid ICMM-CSIC Spain; i Department of Chemistry, University College London UK; j Réseau sur le Stockage Electrochimique de l’Energie (RS2E), CNRS FR 3459 80039 Amiens Cedex 1 France; k Department of Chemistry, Merkert Chemistry Center, Boston College Chestnut Hill MA USA

## Abstract

Since its verification in 2019, there have been numerous high-profile papers reporting improved efficiency of lithium-mediated electrochemical nitrogen reduction to make ammonia. However, the literature lacks any coherent investigation systematically linking bulk electrolyte properties to electrochemical performance and Solid Electrolyte Interphase (SEI) properties. In this study, we discover that the salt concentration has a remarkable effect on electrolyte stability: at concentrations of 0.6 M LiClO_4_ and above the electrode potential is stable for at least 12 hours at an applied current density of −2 mA cm^−2^ at ambient temperature and pressure. Conversely, at the lower concentrations explored in prior studies, the potential required to maintain a given N_2_ reduction current increased by 8 V within a period of 1 hour under the same conditions. The behaviour is linked more coordination of the salt anion and cation with increasing salt concentration in the electrolyte observed *via* Raman spectroscopy. Time of flight secondary ion mass spectrometry and X-ray photoelectron spectroscopy reveal a more inorganic, and therefore more stable, SEI layer is formed with increasing salt concentration. A drop in faradaic efficiency for nitrogen reduction is seen at concentrations higher than 0.6 M LiClO_4_, which is attributed to a combination of a decrease in nitrogen solubility and diffusivity as well as increased SEI conductivity as measured by electrochemical impedance spectroscopy.

## Abstract 2

Since its verification in 2019, there have been numerous high-profile papers reporting improved efficiency of lithium-mediated electrochemical nitrogen reduction to make ammonia.

## Graphical abstract

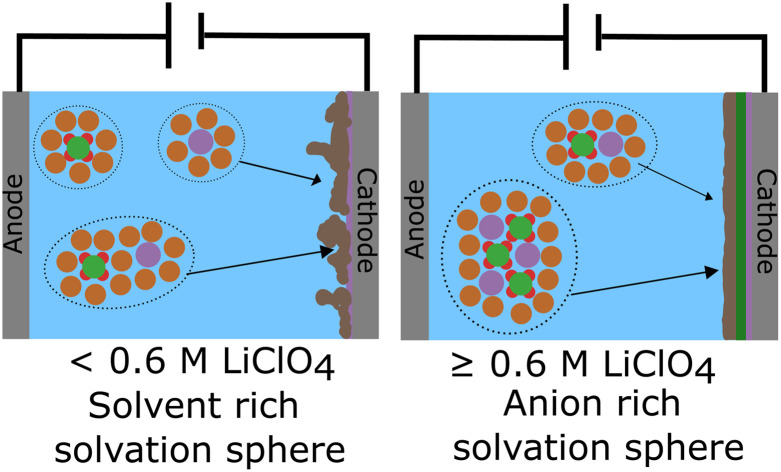

**10th anniversary statement**

We would like to congratulate the Journal of Materials Chemistry A on their 10 year anniversary. The journal has been instrumental in bringing the materials research community together, especially in the fields of energy and sustainability, and has highlighted major breakthroughs in the area. In our paper on electrochemical ammonia synthesis, we utilise the wealth of information on battery science to gain insight into lithium-mediated nitrogen reduction. In particular, we demonstrate the importance of Solid Electrolyte Interphase (SEI) tailoring and understanding, drawing on the recognised effect of salt concentration on SEI stability. We hope that this work, among others, promotes further interdisciplinary investigation with the aim of moving beyond lithium. Prof. Magda Titirici states “It was such a joy and privilege for me to act as an associate editor over the past 10 years, and to see the journal flourish. I have published and reviewed many battery papers in J. Mater. Chem. A, and it is wonderful to be involved in this work translating my battery knowledge to electrocatalysis”. Dr Ifan Stephens states “It is particularly pertinent that our paper is published in J. Mater. Chem. A, as it draws on battery science to understand catalysis; over the past 10 years, the journal has spearheaded high quality research in both fields”.

## 1. Introduction

Ammonia is one of the highest value chemicals currently produced, with the advent of the Haber–Bosch process to make ammonia in the early 20th century allowing for bulk production of fertiliser.^1^ Ammonia also has potential for implementation as an energy dense, readily liquified carbon-free fuel.^1^ However, whilst well-optimised and efficient, the Haber–Bosch process presents a significant environmental challenge. To produce the current annual yield of 175 Mt of Haber–Bosch ammonia,^2^ extreme pressures (>150 bar) and temperatures (>400 °C) are required to provide favourable thermodynamics and kinetics.^3^ This limits Haber–Bosch ammonia production to large, centralised plants to be efficient,^4^ resulting in logistical and financial issues with ammonia supply.^5^ In addition, the hydrogen required for the process is primarily sourced from methane steam reforming, which releases huge amounts of CO_2_. Haber–Bosch ammonia production therefore generates approximately 1% of global greenhouse gas emissions^6^ and results in the consumption of ∼1% of global energy requirements.^7^

A better solution would be one powered by electricity from renewable energy sources operating at ambient temperature and pressure, which would eliminate carbon emissions. Such a solution would also allow for production at the point of use, reducing capital expenditure and global fertiliser inequity.^5,8^ There has been, therefore, a great deal of interest over the past 30 years in electrochemical nitrogen reduction. Here, N_2_ gas is reduced on a catalyst surface in the presence of protons to produce ammonia. However, the vast majority of results are false positives.^9^ It is likely impossible to efficiently reduce nitrogen to ammonia in aqueous electrolytes due to extreme competition with the hydrogen evolution reaction (HER), which can also result in electrode poisoning and deactivation.^10,11^ The only rigorously verified electrochemical nitrogen reduction paradigm is that pioneered by Tsuneto *et al.* in the 1990s and later verified by Andersen *et al.* in 2019.^12–14^ This is the lithium-mediated nitrogen reduction system, where an organic solvent, non-aqueous proton source and lithium salt work in concert to allow for non-negligible ammonia yields. Since 2019, great steps forward have been taken in system optimisation by considering cell design and choice of lithium salt,^3,15–18^ proton source choice,^13,16,19^ N_2_ partial pressure,^13,19,20^ potential cycling,^20^ and oxygen inclusion.^21^ However, while these advances have resulted in commendable improvements in selectivity, stability and activity, there has been little to no investigation into exactly *why* the lithium mediated system is able to outperform all other solid-electrode paradigms.^22^

Most models cite the ability of lithium metal – which is plated on the working electrode *in situ* – to spontaneously dissociate the highly energetic N_2_ triple bond as the driving force of the reaction.^3,20^ However, this strong binding to N_2_ is accompanied by a stronger binding to protons;^23^ this is problematic given the requirement of the presence of both N_2_ and protons to form ammonia. Furthermore, when considering the sole example of efficient, stable and active ambient nitrogen reduction, the enzyme nitrogenase, it is clear that immediate N≡N scission is not a pre-requisite for ammonia synthesis.^24,25^ This phenomenon is echoed in homogeneous systems.^26,27^ The key to the lithium-mediated system could lie in lithium's unique ability to form a Solid Electrolyte Interphase (SEI).^22^ On the first charging cycle of a Lithium-ion Battery (LiB), electrolyte decomposition products are deposited onto the electrode surface. These form a layer that is electronically insulating but lithium ion conducting, providing kinetic stability to prevent further electrolyte degradation.^28^ It is the formation of this SEI layer that allows LiBs with graphite anodes to operate for 1000 s of cycles. A similar layer is formed in the lithium-mediated nitrogen reduction paradigm, as evidenced by Electrochemical Impedance Spectroscopy (EIS),^29^ X-Ray Photoelectron Spectroscopy (XPS) and X-Ray Diffraction (XRD) measurements.^21^ The SEI layer likely controls the access of protons to the electrode surface, reducing competition with the HER and allowing the system to make ammonia.^20,22,30^ Herein, we rigorously link bulk electrolyte properties to SEI characteristics to fully understand their impact on the subsequent efficiency of nitrogen reduction, understanding which has thus far been lacking in the literature.

**Fig. 1:**
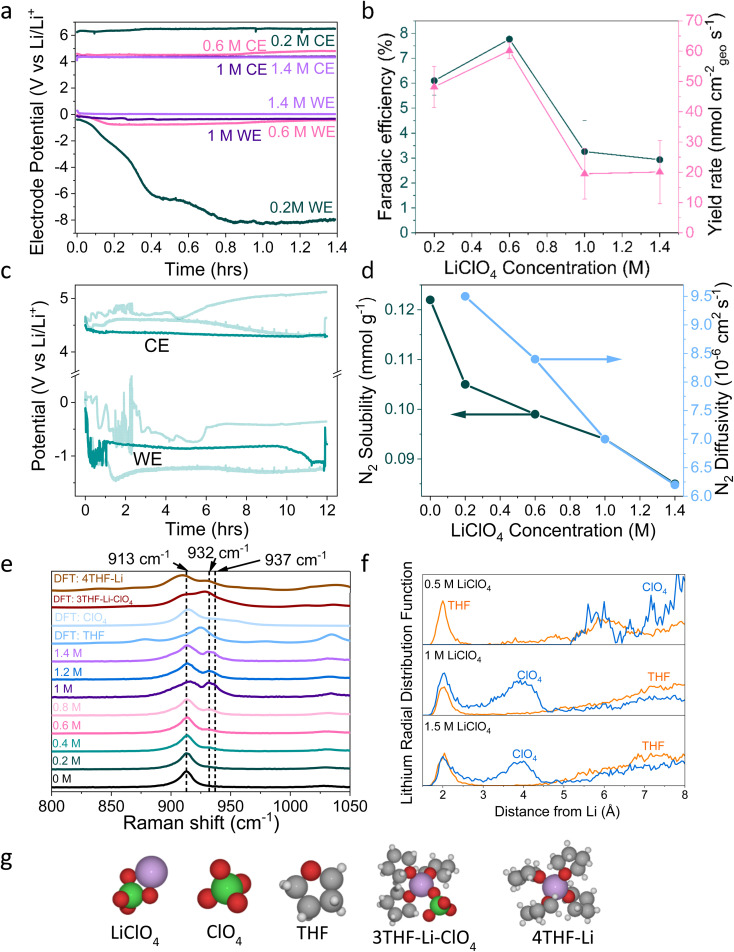
(a) The change in working electrode (WE, molybdenum foil coated with *in situ* deposited Li_*x*_N_*y*_H_*z*_) stability with LiClO_4_ concentration. A constant current density of −2 mA cm^−2^ is applied until −10C is passed. Stability occurs at 0.6 M LiClO_4_, where the counter electrode (CE, platinum mesh) potential also settles at a lower value. WE and CE potentials reported *vs.* the observed lithium plating potential and corrected for ohmic losses. A Pt wire is used as a pseudo-reference. The electrolyte is varying concentrations of LiClO_4_ in THF containing of 1% v/v EtOH as a sacrificial proton donor. Further experimental details can be found in the ESI (Fig. S2–S4†) (b) The change in faradaic efficiency and yield rate with LiClO_4_ concentration (*n* = 3 separate experiments, error bar is standard error in the mean) for a chronopotentiometry experiment at an applied constant current of −2 mA cm^−2^ until −10C is passed. (c) The extended operation of a 0.6 M LiClO_4_ electrolyte. Potential reported *vs.* the observed lithium plating potential and corrected for ohmic losses. Two other greyed out traces are shown to indicate the reproducibility of the experiment. −2 mA cm^−2^ was applied for 12 hours. (d) The change in N_2_ solubility and diffusivity in THF at different concentrations of LiClO_4_. Solubility and diffusivity were measured using N_2_ absorption with a porosity analyser. See ESI section 8 and Fig. S7† for full experimental details. (e) Simulated and experimental Raman spectra of various co-ordination geometries of LiClO_4_ in THF. Simulated spectra are obtained using Density Functional Theory (DFT). See ESI part 9† for details on DFT calculations. (f) Theoretical Radial Distribution Functions (RDFs) for lithium in 0.5 M, 1 M and 1.5 M LiClO_4_ concentrations in THF. The RDFs were obtained using *ab initio* molecular dynamics, as explained in ESI part 9.† (g) Space filling diagrams of LiClO_4_, THF, ClO_4_^−^ and the 4THF–Li and 3THF–Li–ClO_4_ clusters. DFT data set can be found in ref. 31.

A well-established issue with the electrolyte originally used by Tsuneto *et al.,*^13,14^ and since employed by Chorkendorff, Vesborg, Nørskov and coworkers,^12,20^ is its lack of stability under the required electrochemical conditions. At the beginning of a chronopotentiometry experiment, where a constant current density is applied and the electrode potential measured, the working (negative) electrode sits at lithium plating potentials. However, over the course of the experiment, the working electrode becomes more negative compared to the counter (positive) electrode (Fig. 1a and S6†). This problem is henceforth referred to as ‘working electrode drift’, and results in significantly decreased energy efficiency.^18,19^ This electrolyte is made of up 0.2 M LiClO_4_ dissolved in tetrahydrofuran (THF), with an addition of 1% v/v EtOH as a sacrificial proton donor, henceforth referred to as the Tsuneto electrolyte. Clearly this problem and the resulting poor energy efficiency means that the Tsuneto electrolyte is not suitable for any realistic device.

A variety of solutions to the problem of working electrode drift have been presented in the literature, but there has been no clear explanation as to exactly where the problem stems from. Chorkendorff, Vesborg, Nørskov and coworkers addressed working electrode drift by using a potential cycling method, where a short current pulse of 2 mA cm^−2^ was applied, followed by a period at open circuit potential. Here the authors claim the pulsing technique prevents the build-up of undesired products on the working electrode.^20^ Suryanto *et al.* achieved improved stability through electrolyte design, utilising a phosphonium salt as a recyclable proton donor, in place of sacrificial ethanol, and 0.2 M LiBF_4_ rather than 0.2 M LiClO_4_, but do not explain the origin of their improved stability.^19^ Chorkendorff, Vesborg, Nørskov and coworkers^21^ have also shown that the introduction of small amounts of oxygen into the inlet gas results in greater stability, citing increased SEI homogeneity and decreased Li^+^ ion diffusion as reasons for higher faradaic efficiency and stability. Du *et al.* also achieved close to 100% faradaic efficiencies using higher concentrations of LiNTf_2_ salt under 15 bar N_2_, suggesting that a thin and dense SEI results in improved Li cycling and faradaic efficiency.^17^ However, this study did not focus on SEI or bulk electrolyte characterisation. It is also worth stating that the relationship between faradaic efficiency and N_2_ partial pressure is relatively well understood; the faradaic efficiency increases with increasing N_2_ partial pressure up to a certain point, after which it no longer improves. Andersen *et al.* note this point as 10 bar N_2_ partial pressure.^20^ Chorkendorff, Nørskov, Vesborg and co-workers recently show that the use of a fluorinated salt results in the formation of a LiF layer, which improves SEI stability and electrochemical performance.^18^ However, the reason why the Tsuneto electrolyte offers poor stability remains a relative mystery. In this work we seek to fully understand the problem of working electrode drift in relation to bulk electrolyte and SEI properties, as well as to provide further fundamental insight into the mechanisms of lithium mediated nitrogen reduction.

Our initial studies suggest that the SEI formed in the Tsuneto electrolyte may either be unstable or not fully passivating, with decomposition products continuing to be deposited after initial cycling. Time-of-Flight Secondary Ion Mass Spectrometry (ToF-SIMS) data in Fig. S1† shows that the SEI becomes more dominated by heavy, likely organic mass fragments after the electrode potential has been allowed to drift. Such organic species are likely to result from continued solvent decomposition.^32^ We hypothesise that this increase in organic species results in a more resistive SEI, forcing the electrode to more negative potentials to be able to continue passing the same constant current density. The answer to this issue can be found in battery science.

The electrolytes used in LiBs have been well optimised. The most common choice is a mixture of 1 M LiPF_6_ in an organic solvent containing a cyclic carbonate, often ethylene carbonate (EC), as an SEI-former, and a linear carbonate, often dimethyl carbonate (DMC), to maintain the optimal physical properties of the electrolyte.^33^ Several factors govern this choice, one of which is to provide an optimal battery SEI.^33^ It is important to note, however, that the ideal N_2_ reduction SEI may differ from the ideal battery SEI.^21^ The properties of the SEI are primarily tailored by varying the composition of the bulk electrolyte, both *via* the ratio of solvents used and by the addition of electrolyte additives such as vinylene carbonate (VC) or fluoroethylene carbonate (FEC).^34^ The SEI is strongly affected by the solvation environment of the Li^+^ ion, since whatever is contained within the Li^+^ solvation shell will be preferentially reduced on the electrode on the first charging cycle. If the Li salt is well solvated by the solvent, the solvation shell will be made up of more solvent molecules than salt anions, which will result in an SEI made up primarily of organic solvent decomposition products.^32^ This is similar to what we see in Fig. S1† when using the Tsuneto electrolyte. However, in the burgeoning field of superconcentrated electrolytes, very high concentrations of lithium salt in an organic solvent can be used to increase the stability of the SEI, especially for lithium-metal batteries.^33^ This phenomenon is possible since the high concentration of salt increases the lithium salt anion content in the Li^+^ solvation shell, which creates a SEI layer that is mostly comprised of inorganic salt reduction products.^35^ These themes from battery science can provide insight for the Li-mediated nitrogen reduction system. Increasing the concentration of salt in the Tsuneto electrolyte could create a more stable, more inorganic SEI and prevent working electrode drift, which was briefly mentioned by Li *et al.*,^36^ where the authors noticed an improvement in stability with a 2 M LiClO_4_ concentration. Until now there has been limited fundamental understanding of exactly how the bulk characteristics of the electrolyte impact the catalytic performance of the system, and even less understanding into the role or chemical makeup of the SEI.

In this paper, we use a model system, the Tsuneto electrolyte under 1 bar N_2_ partial pressure, to study the effect of increased salt concentration on faradaic efficiency and SEI characteristics in detail. While other studies have made excellent progress in improving stability, faradaic efficiency and activity,^18,22^ here we seek to gain more fundamental understanding of the exact properties of the bulk electrolyte and SEI which yield the best results. We link bulk electrolyte properties, such as salt solvation and N_2_ solubility and diffusivity, to SEI characteristics, system stability, ammonia yield and faradaic efficiency using a combination of electrochemical measurements, Density Functional Theory (DFT) calculations, bulk electrolyte characterisation and a variety of advanced post-mortem characterisation techniques.

## 2. Electrochemical results

Fig. 1a shows electrochemical data from chronopotentiometry experiments at a constant applied current of −2 mA cm_geo_^−2^. At the working electrode, nitrogen is reduced to form ammonia. The counter electrode reaction is not controlled, but likely involves solvent oxidation, as previously documented in the field.^18,37,38^ A change in working electrode potential stability is observed upon changing the LiClO_4_ concentration from 0.2 M to 1.4 M. A transition to much improved stability is observed at 0.6 M, coinciding with a maximum faradaic efficiency of 7.8 ± 0.5% and yield rate of 60 ± 3 nmol cm^−2^ s^−1^ (*n* = 3) (Fig. 1b). At higher concentrations, stability is maintained but faradaic efficiency decreases. It is likely that the rest of the current density goes towards a combination of lithium plating, hydrogen evolution and continued SEI formation in varying proportions at different salt concentrations.^13,14,20^ Fig. S5† highlights the reproducibility of this trend. The 0.6 M electrolyte is also relatively stable over a time period of 12 hours (Fig. 1c), although the average obtained faradaic efficiency is lower at 4.7 ± 0.5% (*n* = 3). This lower obtained faradaic efficiency may be due to electrolyte evaporation over the course of a 12 hour experiment causing the salt concentration to increase (see Table S1†), as well as consumption of ethanol changing the proton donor concentration over the course of a longer experiment.

Interestingly, the counter electrode potential is also affected by the salt concentration. At 0.2 M LiClO_4_, the potential remains stable at approximately 6 V *vs.* Li^+^/Li, whereas the more concentrated samples all have less positive counter electrode potentials at around 4 V *vs.* Li^+^/Li.^37^ The impact of the anode reaction on the lithium-mediated nitrogen system will be the subject of further studies. Fig. S6† shows the change in the DFT-calculated HOMO–LUMO (Highest Occupied Molecular Orbital–Lowest Unoccupied Molecular Orbital) of THF at various concentrations of LiClO_4_. As LiClO_4_ concentration increases, the HOMO–LUMO separation decreases. HOMO–LUMO separation is not directly related to electrolyte stability, and instead electrolyte redox potentials should be considered. However, in many cases the redox potentials of non-aqueous solvents are correlated with the HOMO–LUMO gap.^39^ Therefore, as we increase salt concentration, we may decrease the thermodynamic electrolyte stability by closing the HOMO–LUMO gap, which may partly explain the change in anode potential. However, the formed SEI in the more concentrated electrolytes appears to be more able to kinetically stabilise the system at the cathode due to the observed increase in working electrode stability in Fig. 1a and S5.†

## 3. Bulk electrolyte characterisation

Fig. 1d shows the decrease in N_2_ solubility and diffusivity with increasing LiClO_4_ concentration. Details on experimental procedures for determining N_2_ solubility can be found in the ESI.† This decreasing N_2_ solubility and diffusivity may partially explain the drop in faradaic efficiency observed for LiClO_4_ at concentrations greater than 0.6 M. In a diffusion limited generalised lithium-mediated nitrogen reduction system, Andersen *et al.*^20^ propose that, in excess of dissolved nitrogen, the rate of ammonia production becomes limited by the rate of proton transfer to the active surface. However, reducing the availability of dissolved nitrogen leads to an excess of protons, which results in the partial current density towards ammonia being driven by the rate of nitrogen transfer to the surface. Given that the cathodic current for this system can be assumed to be dominated by hydrogen evolution, we can assume that there is an excess of protons at the electrode surface. Therefore, increasing N_2_ solubility and diffusivity in the bulk electrolyte would allow for a greater partial current density towards ammonia production rather than hydrogen evolution.

Indeed, we can obtain the limiting current density for nitrogen reduction from the equation

1 *J*_lim_ = *nFD*_N_2__[N_2_]*δ*^−1^,

where *n* is the number of electrons involved in the rate limiting reaction, *F* is the Faraday constant, *D*_N_2__ is the diffusion constant, [N_2_] is the molar concentration of N_2_ and *δ* is the Nernst diffusion layer thickness.^40^Eqn (1) shows a first order dependence on both solubility and diffusivity for the limiting current density, and so the monotonic decrease in solubility and diffusivity with increasing salt concentration will result in a decrease in limiting current density. Indeed, assuming a constant Nernst diffusion layer thickness, we can calculate that increasing the salt concentration from 0.6 M to 1.4 M decreases the limiting current density by approximately 40%, which roughly corresponds to the observed decrease in faradaic efficiency of approximately 60%. This problem could be mitigated by operating at higher N_2_ partial pressure, hence increasing N_2_ solubility, and could also partly explain the lower faradaic efficiency achieved by Li *et al.* when operating at 2 M LiClO_4_.^36^ We can also rule out increasing water content with salt content as a cause of the reduction in faradaic efficiency at higher salt concentration; Table S2† shows typical water contents in the different electrolytes, and there is not a significant increase in water content with salt content.

Fig. 1e shows simulated (labelled DFT) and experimental Raman data for varying concentrations of LiClO_4_ in THF and different THF–Li–ClO_4_ environments, shown in space filling diagrams in Fig. 1g. In the experimental 0 M LiClO_4_ spectrum, we assign the central peak at 913 cm^−1^ to the C–O stretching frequency of THF.^41^ As LiClO_4_ concentration increases, a shoulder is formed to the right of the central THF peak, broadening with increasing concentration to include two peaks at 932 cm^−1^ and 937 cm^−1^. Kameda *et al.* note that the symmetric stretching mode of ClO_4_^−^ overlaps with the C–O stretching mode of THF and is also strongly affected by interactions with Li^+^ ions.^42,43^ This effect causes a shift to higher wavenumbers with increasing interaction, so the shift is weakest for free solvated ions, moderate for Solvent Separated Ion Pairs (SSIPs) and for Contact Ion Pairs (CIPs), and strongest for aggregates.^42^ SSIPs are a pair of ions separated by a solvent molecule, whereas CIPs are ion pairs not separated by a solvent molecule. Free solvated ions are not connected to another ion at all. There is some confusion in the literature about how to identify the exact solvation environment of lithium perchlorate *via* Raman spectroscopy. Some authors suggest that the free solvated ClO_4_^−^ ion peak occurs at around 933 cm^−1^ ^42^ and the SSIP peak occurs at between 937 cm^−1^ ^42^ and 939 cm^−1^,^41^ with the CIP peak forming at around 948 cm^−1^.^43^ However, later authors suggest that free solvated ions and SSIPs are spectroscopically identical, and so assigned the peaks at around 939 cm^−1^ and 948 cm^−1^ to CIPs and aggregates respectively.^43,44^ This makes it difficult to determine the exact solvation environment of the Li^+^ ion. Fig. S8† shows Fourier Transform Infra-Red (FTIR) spectra obtained for varying LiClO_4_ concentrations in THF. These spectra suggest the formation of more highly coordinated ion environments with increasing salt concentration, with evidence for the presence of both CIPs and aggregates.^44^ In Fig. 1e, we therefore assign the 932 cm^−1^ peak to free solvated ClO_4_^−^ or SSIPs, and the 937 cm^−1^ peak to more highly coordinated geometries, such as CIPs or aggregates.

The DFT simulated spectra in Fig. 1e of the 4THF–Li and 3THF–Li–ClO_4_ clusters also show the change in the oxygen–lithium bonding environment, either between oxygen from THF and lithium, or oxygen from ClO_4_ and lithium. The main peak for 4THF–Li occurs at around 910 cm^−1^, which may explain the stretching of the central THF peak towards lower wavenumbers due to an increasing lithium concentration. There is a peak at around 930 cm^−1^ in the simulated 3THF–Li–ClO_4_ environment that may correspond to fully solvated ClO_4_^−^ or an SSIP. Whilst the simulated Raman spectra are not a one-to-one match with the experiments on Raman-shift, they do indicate how the Li–ClO_4_ clusters pair with THF molecules to introduce the additional right-hand peak at higher salt concentrations.

Fig. 1f shows the DFT calculated Radial Distribution Functions (RDF) of a lithium ion in THF with increasing LiClO_4_ concentration. As LiClO_4_ concentration increases, it becomes more likely to find an oxygen from the ClO_4_^−^ anion within the first few angstroms from the lithium. Therefore, as the LiClO_4_ concentration increases, the average interaction between the oxygen from the ClO_4_^−^ and Li will get stronger due to increased proximity, resulting in the peaks seen in the experimental Raman data and the formation of more coordinated solvation structures.

## 4. SEI characterisation

**Fig. 2:**
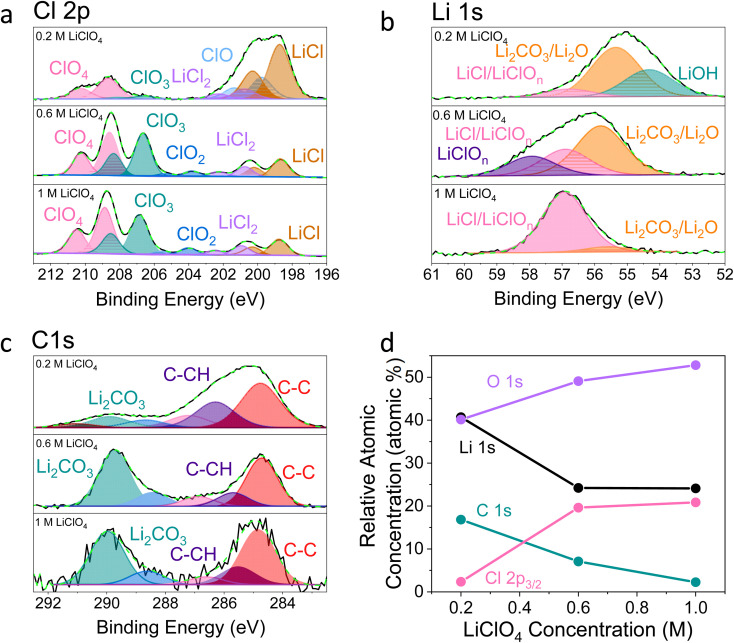
X-ray photoelectron spectroscopy spectra of a Cu working electrode after passing 10C at −2 mA cm^−2^ under N_2_ for 0.2 M, 0.6 M and 1 M LiClO_4_ in 99 : 1 THF : EtOH electrolyte. All spectra are normalised to the maximum value for that spectrum. Therefore, all intensities are relative rather than absolute. (a) Cl 2p, (b) Li 1s, (c) C 1s, (d) how the relative atomic concentration of O, Li, C and Cl change as electrolyte salt concentration varies according to XPS. The O 1s core level had no clear features (see Fig. S9†). The N 1s core level was too low intensity to be observable.

**Fig. 3:**
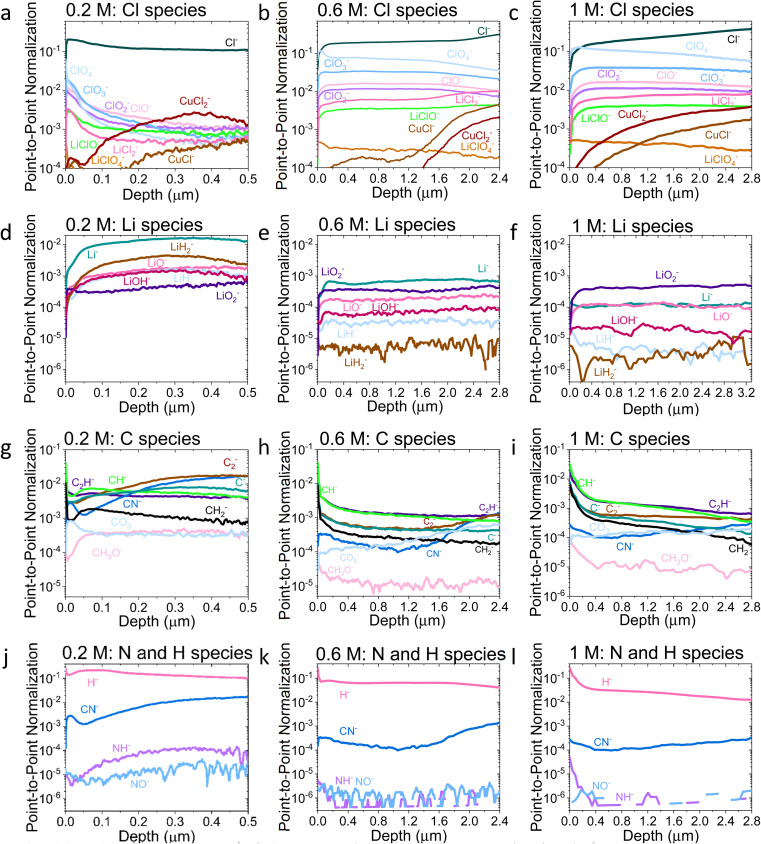
Comparison of the ToF-SIMS traces for the 0.2 M, 0.6 M, and 1 M LiClO_4_ samples on a Cu working electrode after passing −10C under N_2_ at −2 mA cm^−2^. (a–c) Cl species for 0.2, 0.6, and 1 M samples, (d–f) Li species for 0.2, 0.6, and 1 M samples, (g–i) C species for 0.2, 0.6, and 1 M samples and (j–l) N and H species for 0.2, 0.6, and 1 M samples. All traces normalised to total counts point-to-point. The traces are shown from the surface of the SEI (0 μm) to the Cu surface of that sample. Sputtering was done with Ar^+^ clusters (*n* = 1159). Depth is estimated using the crater depth and assuming a constant sputter rate. Crater depth was measured using an optical interferometer. Full experimental details can be found in the ESI section 4b.†

**Fig. 4:**
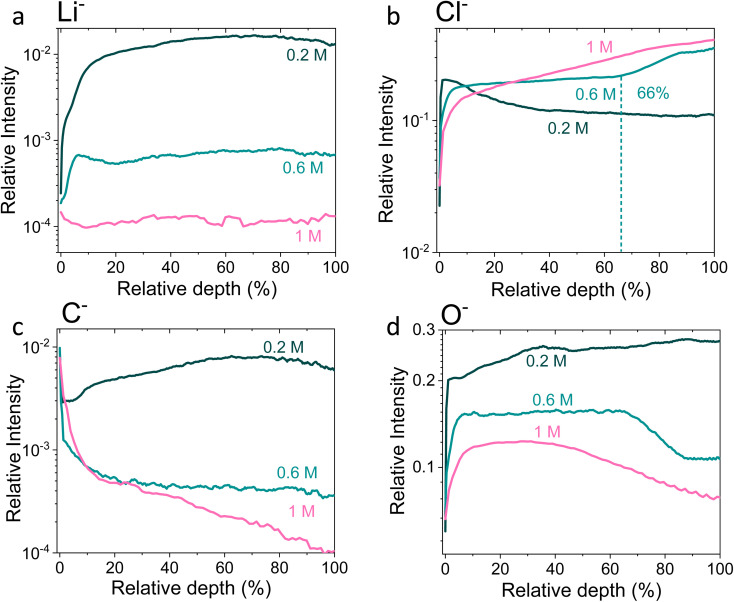
Time-of-flight secondary ion mass spectrometry depth profiles of a Cu working electrode after passing 10C at −2 mA cm^−2^ under N_2_ for 0.2 M, 0.6 M and 1 M LiClO_4_ in 99 : 1 THF : EtOH electrolyte. All intensities are normalised to total counts point-to-point. The relative depth parameter represents the depth through the SEI, where 0% is the surface of the sample and 100% is the completed removal of the SEI. The 0.2 M SEI sample was approximately 0.5 μm thick, the 0.6 M SEI sample was approximately 2.8 μm thick and the 1 M SEI sample was approximately 3.3 μm thick. (a) Shows the change in the Li^−^ signal with relative depth, (b) shows the change in Cl^−^ signal with relative depth, (c) shows the change in O^−^ signal with relative depth, and (d) shows the change in C^−^ signal with relative depth. Sputtering was done with Ar_*n*_^+^ clusters (*n* = 1159), which is more gentle than single Ar^+^ ions. Full experimental details can be found in the ESI section 4b.†

Fig. 2–4 show post-mortem XPS spectra and ToF-SIMS traces collected for three SEI samples formed using either a 0.2 M, 0.6 M, or 1 M LiClO_4_ electrolyte. In this case, the same experimental procedure was carried out as for the electrochemical investigations in Fig. 1, but a copper working electrode was used to avoid the overlap of the Mo 3p and N 1s core levels in XPS. Here we use XPS to provide information about the chemical environment at the SEI surface and ToF-SIMS to reveal complementary information about the change in chemistry with depth into the SEI (note that the depth profiling experiments are carried out using Ar^+^ ion clusters for sputtering to minimize sample damage).^38^

It is clear from Fig. 2 that the surface chemistry shown in the 0.2 M LiClO_4_ XPS spectra differs significantly from those of the 0.6 and 1 M samples. The organic contribution is much higher for the 0.2 M sample, shown in Fig. 2c and d, which skews the results for the other core level spectra. This echoes our initial ToF-SIMS data (Fig. S1†) which show that the organic content of the 0.2 M LiClO_4_ SEI increases with time spent at constant current, likely causing total SEI resistance to increase and the working electrode potential to drift to more negative values. However, is also likely that the SEI morphology differs between the three samples. If the 0.2 M sample is more organic, it is likely to be more porous than the higher concentration samples. This may result in a higher contribution from the outermost layers in XPS. Since the information depth of XPS is only a few nanometres, a larger proportion of the measured signal would be from the very surface rather than inside the SEI in a more porous sample. This increased contribution from the SEI surface for the 0.2 M sample compared to the 0.6 and 1 M samples would cause a higher measured level of total carbon and levels of oxidation and hydroxylation, which can be observed in the possible presence of an LiOH peak in the Li 1s core level of the 0.2 M sample (Fig. 2b). Fig. 2d shows the relative atomic concentration of the four XPS core levels considered. The Li 1s and C 1s core levels decrease compared to the O 1s and Cl 2p core levels with increasing LiClO_4_ concentration, dramatically between 0.2 and 0.6 M, and then more slowly from 0.6 to 1 M. The dramatic difference between the surface chemistries of the 0.2 M and 0.6 and 1 M samples is in line with what we observe in the electrochemical data; while the 0.2 M sample experiences working electrode drift, the 0.6 M and 1 M samples do not. Preliminary microscopy images (not shown) may also show evidence of a more porous morphology for the 0.2 M SEI. This will be the focus of future work. While we can still draw interesting comparisons between the surface chemistry of the three samples, it is important to keep these caveats in mind.

Fig. 2a shows that the relative LiCl to ClO_4_ content of the 0.2 M sample is much higher compared to the 0.6 and 1 M samples shown in Fig. 2b and c. This could be due to the presence of LiClO_4_ on the surface of the SEI from the electrolyte in samples from the more concentrated solutions, which remained despite washing; more highly concentrated electrolytes would result in greater quantities of salt on the electrode surface. It may also be that the washing step removed more of the surface layer for the 0.2 M sample, which could have revealed some more reduced Cl species than for the other samples. Fig. 3a–c show the difference in the relative intensities of the ClO_*x*_^−^ fragments through the SEI for each sample. For the 0.2 M sample, the ClO_*x*_^−^ fragments sharply decrease in relative intensity with depth, whereas the 0.6 and 1 M samples have a more consistent ClO_*x*_^−^ depth profile. This phenomenon can also be explained by considering the Raman spectra and radial distribution functions shown in Fig. 1d and e. As salt concentration is increased, the likelihood of finding the ClO_4_^−^ anion in the Li^+^ solvation shell increases. Thus, it becomes likely that the ClO_4_^−^ anion will penetrate into the SEI more deeply and in greater quantities, along with products related to its decomposition. Therefore, the ClO_4_^−^ anion and related inorganic decomposition products are more abundant in the SEI formed in more concentrated electrolytes. This likely explains the increase in working electrode stability at higher electrolyte concentrations.^34^

Although Li 1s core levels are notoriously difficult to fit due to the small chemical shift between different Li species, Fig. 2b shows that, as concentration increases, the Li 1s spectrum becomes more symmetrical and decreases in width. This suggests fewer Li species present at the surface of the SEI with increasing concentration, perhaps leaving only lithium bound to ClO_*n*_^−^ species. Additionally, only the 0.2 M sample shows a low binding energy contribution at 54.31 eV in the Li 1s spectrum, possible evidence of the presence of LiOH. We expect that all three samples should contain some LiOH due to reactions with trace water^28,45^ (Table S2†). Indeed, the ToF-SIMS data in Fig. 3d–f show the presence of an LiOH^−^ fragment in all three samples. This could suggest that the potential oversampling of the surface in XPS of the 0.2 M sample lead to greater signal from the LiOH chemical environment in XPS. However, ToF-SIMS is unable to provide quantitative information, so it is difficult to determine if the amount of LiOH at the surface of the more concentrated samples is enough to be detectable in XPS. It may be that the SEI in the more concentrated electrolytes provides greater protection against reactions between lithium and water. Further investigation is required to confirm this hypothesis.

Fig. 2c shows the C 1s core levels obtained for the three SEI samples. The chemical composition of the carbon species does not change appreciably between samples, but the relative intensity of the signal does decrease (highlighted by the increased noise in the more concentrated samples and Fig. 1d). Indeed, Fig. 3g–i do not reveal any different carbon containing species between the three samples. This is to be expected, since organic SEI components will result from solvent decomposition, which remains constant between the three samples.

Both the XPS and ToF-SIMS data did not reveal the presence of many nitrogen containing species within the limits of detection. The N 1s core level was not detectable in the XPS data, and only three nitrogen containing species were observed in ToF-SIMS: NH^−^, NO^−^ and CN^−^ (Fig. 3j–l). Critically, no lithium–nitrogen species were observed, such as fragments of a mixed Li_*x*_N_*y*_H_*z*_ species that were predicted as the catalytically active surface by Schwalbe *et al.*^29^ or fragments of Li_3_N as reported by Li *et al.*^21^ The absence of such species may be because a large Li_*x*_N_*y*_H_*z*_ molecule is likely to be broken up into smaller fragments during ToF-SIMS analysis, or because only negative ions were collected. LiH^−^ and LiH_2_^−^ are observed (Fig. 3d–f), which may be possible fragments of Li_*x*_N_*y*_H_*z*_, but there is not an obvious correlation in relative intensity with any N containing species. Li *et al.* reported Li_3_N as a surface but not a bulk species. The authors used a 0.3 M LiClO_4_ in 99 : 1 THF : EtOH electrolyte, similar to that used in this study, but operated under 20 bar N_2_ and at varying O_2_ concentrations, which may alter the surface chemistry. Since the authors generated a much greater quantity of ammonia when operating under these conditions than in this work, it would make sense that a greater quantity of nitrogen containing species were observable.^21^ However, from the ToF-SIMS data in our current work in Fig. 3, any nitrogen at the surface of the SEI here is more likely to be some nitrogen containing organic species. It may be that these species form from a reaction between an intermediate of nitrogen reduction and the organic electrolyte; interestingly the CN^−^ trace increases in intensity with depth for all three SEI samples (Fig. 3j–l).

Indeed, the detection of reaction intermediates is likely to be difficult *via* an *ex situ*, post-mortem technique. While Li_3_N is stable in the absence of protons, with a standard Gibbs free energy of formation of −154.8 kJ mol^−1^,^46^ in the presence of a proton source it should rapidly decompose to form ammonia.^29^ LiH is slightly less thermodynamically stable with a standard Gibbs free energy of formation of −68.3 kJ mol^−1^,^47^ but is less likely to decompose in the presence of protons, which could explain why we are able to observe the lithium-hydrogen species. Additionally, Fig. 3d–f do not show any evidence of a layer composed solely of lithium. It is likely that any metallic lithium surface would have reacted to form something else. Further *in situ*, operando experiments will be required to probe the active catalytic site of ammonia production.

Fig. 3a–c show an increase in the relative intensity of the CuCl^−^ and CuCl_2_^−^ traces with depth through the SEI, suggesting an interaction of the copper electrode with the electrolyte, with the rise in the 1 M sample being steadier than that of the 0.2 and 0.6 M samples. In LiBs, transition metal dissolution in current collectors presents a significant stability issue^48^ which generally increases with increasing salt concentration.^49^ It is therefore likely that the more concentrated electrolytes result in a greater level of degradation in the Cu electrode, which could lead to more Cu containing species in the SEI. It is likely that this problem is less severe for the Mo electrodes; Cu creates a native oxide in air which is easily removed under reducing potentials,^48^ whereas we anticipate that of Mo is kinetically challenging to reduce. Nonetheless, the choice of electrode between Cu and Mo does not seem to greatly affect the faradaic efficiency of the system (Table S1†), and the Cl–Cu traces shown in Fig. 3a–c are, in general, of lower relative intensity than the other Cl containing traces, except for the 0.2 M LiClO_4_ sample. For this sample, all Cl containing traces are of quite low relative intensity after a depth of about 0.25 μm.

The ToF-SIMS data also show a change in the SEI thickness with salt concentration. By evaluating the time at which the Cu^−^ signal reached a peak (Fig. S10†), it is possible to estimate the thickness of the SEI using the total crater depth through optical interferometry. The 0.2 M, 0.6 M and 1 M LiClO_4_ SEI samples were approximately 0.5 μm, 2.8 μm, and 3.3 μm thick respectively, assuming a constant rate of sputtering. Note that this assumption does introduce some errors and the sputter rate may not be homogeneous throughout different parts of the SEI and the Cu electrode, and each will have different densities, but it can provide a good guide of the *relative* thickness of each sample.

Fig. 4 shows the Li^−^, Cl^−^, O^−^ and C^−^ traces obtained *via* ToF-SIMS as representative of the total lithium, chlorine, oxygen and carbon content throughout the SEI. To enable easier comparison between samples, this figure considers relative depth through the SEI as a percentage value, with 0% being the SEI surface and 100% being the surface of the copper electrode. This figure allows us to observe the difference in the depth heterogeneity of the three SEI samples. While the 0.6 M and 1 M LiClO_4_ samples are clearly more organic towards the surface of the SEI and more chlorinated closer to the copper electrode, the 0.2 M LiClO_4_ sample is generally more homogeneous throughout the depth of the SEI. Both the 0.6 M and 1 M LiClO_4_ samples also have decreasing relative oxygen content closer to the copper electrode, whereas the 0.2 M LiClO_4_ sample oxygen content increases with relative depth through the SEI and then saturates approximately 35% of the way through. This could suggest a greater abundance of more reduced species closer to the electrode surface, such as LiCl, in the more concentrated samples. Indeed, for the 0.6 M LiClO_4_ sample Fig. 3b shows an increase in the LiCl^−^ trace intensity can be seen closer to the electrode surface, and the O^−^ and Cl^−^ traces correlate well in Fig. 4b and d. It is also interesting to note the decrease in surface Li_2_CO_3_ content with increasing salt concentration as shown in Fig. 2b. Indeed, Fig. 3g–i show differing behaviour of the CO_3_^−^ fragment with depth between the three samples. For the 0.2 M sample, the relative CO_3_^−^ intensity peaks near the SEI surface before declining and stabilising, whereas for both the 0.6 and 1 M samples, the relative CO_3_^−^ intensity increases with depth through the SEI.

For the Cl^−^ trace in Fig. 4b, we can observe that relative chlorine content increases with depth for the 1 M sample, decreases with depth for the 0.2 M sample and stays relatively stable with depth for the 0.6 M sample until approximately 66% of the way through the SEI when it sharply increases, then saturates (Fig. 4b). This suggests that most of the chlorine in the 0.2 M sample is likely from residual LiClO_4_ from the electrolyte on the surface of the SEI. This can be seen clearly in Fig. 3a. The XPS data in Fig. 2a, however, suggests a greater relative quantity of LiCl than LiClO_4_ at the SEI surface for the 0.2 M sample. However, while the samples were not rinsed for the ToF-SIMS study since we were more interested in bulk information, the samples for XPS were rinsed in 0.1 ml THF to remove dried electrolyte. It may be that the, likely more organic, SEI formed in the 0.2 M LiClO_4_ electrolyte was more soluble in THF and so some of the surface species were washed away to reveal more reduced lithium-chlorine species. It could also be that, since the 0.2 M sample is in general more homogeneous in composition with depth than the other two, as shown in Fig. 3 and 4, we see a greater relative quantity of reduced species at the surface of the SEI than for the 0.6 and 1 M samples.

Thus, these XPS and ToF-SIMS data suggest an increase in SEI stratification, as well as a possible increase in inorganic species, with salt concentration. This suggests that the model of the SEI in battery literature, which consists of a more organic layer close to the electrolyte and a more inorganic layer close to the electrode,^28^ also holds for the SEI formed using the modified Tsuneto electrolyte, but only for the more concentrated LiClO_4_ electrolytes. It also suggests that the more chlorinated layer formed closer to the electrode surface in the 0.6 M and 1 M LiClO_4_ electrolytes is what allows the electrode to become properly protected against further solvent degradation.

**Fig. 5:**
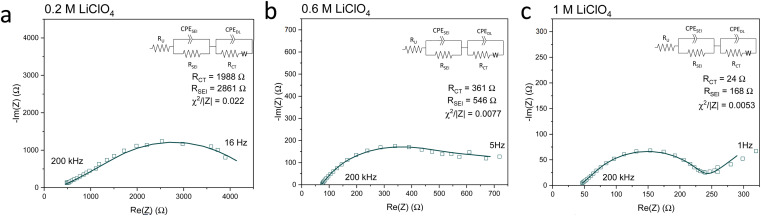
Potentiostatic electrochemical impedance spectroscopy spectra of the same Cu electrodes examined for Fig. 2–4 at the end of a choronopotentiometry experiment. All spectra were recorded between 200 kHz and 100 mHz at an amplitude of 10 mV about open circuit potential, which is approximately 0 V *vs.* Li/Li^+^. In general, the spectra became noisier at lower frequencies so some data points at lower frequencies were omitted. (a) 0.2 M LiClO_4_ electrolyte, (b) 0.6 M LiClO_4_ electrolyte, (c) 1 M LiClO_4_ electrolyte. Fitting parameters shown in ESI Table S3.†

Fig. 5 shows Potentiostatic Electrochemical Impedance Spectroscopy (PEIS) spectra for the 0.2 M, 0.6 M and 1 M LiClO_4_ samples analysed in the XPS and ToF-SIMS. These spectra were fitted to separate the charge-transfer resistance (*R*_CT_) at the SEI-electrolyte interface and the SEI resistance (*R*_SEI_), with a methodology adapted from that of Wang *et al.*^50^ Both *R*_CT_ and *R*_SEI_ decrease with increasing salt concentration. While the exact values of *R*_CT_ and *R*_SEI_ are not reproducible, the general trend is (see Table S3†). Given that the SEI thickness appears to increase with increasing salt concentration, this trend fits with faster kinetics with increased salt concentration. Given that the PEIS spectra give us aggregated information about total charge transfer through the SEI, it is difficult to determine the specific change in Li^+^ ion conductivity with salt concentration. However, both the ToF-SIMS and XPS data in Fig. 2d, 3d–f and 4a show a change in the relative lithium content with increasing salt concentration. While the Li^−^ content continually increases with depth in the 0.2 M sample, the 0.6 M sample exhibits a sharp increase then a plateau and the 1 M sample exhibits no change in relative Li^−^ content. This change in the distribution of Li^−^ in the SEI with increasing LiClO_4_ content could suggest a change in Li^+^ conductivity. In addition, recent DFT studies suggest that Li_2_CO_3_ and Li_2_O allow for fast Li^+^ ion diffusion.^51^Fig. 3d–i show the presence of LiO_2_^−^ for all three samples and increased relative CO_3_^−^ content closer to the electrode surface with increasing LiClO_4_ concentration. This implies the presence of a range of different Li-environments throughout the SEI which change with changing salt concentration. These observations could suggest increased Li^+^ ion conduction in the SEI formed in more concentrated electrolytes, but further investigation is required to be certain of this conclusion.

Chorkendorff, Vesborg, Nørskov and coworkers posit that hindering the transport of Li^+^ ions to the electrode surface can provide a boost to faradaic efficiency, since more electrons will be involved in making ammonia rather than in Li plating.^20,21^ Li *et al.* propose that the inclusion of small quantities of oxygen in their feed gas results in a more homogeneous SEI with reduced Li^+^ transport capability,^21^ citing work by Wang *et al.* which shows that O_2_ inclusion results in improved battery cyclability but increased SEI resistivity.^50^ In the case of the current work, the PEIS (Fig. 5) data points to a decrease in SEI resistance with increased salt concentration. This also supports the hypothesis that, as salt concentration increases, the SEI becomes less organic and so less ionically resistive. This has been shown through the decrease in relative carbon content, and increase in relative chlorine content, in the SEI with increasing salt content in the electrolyte. This is observed *via* XPS (Fig. 2d) and ToF-SIMS (Fig. 3d–i and 4a and b). The increased overall conductivity exhibited by the SEI formed in more concentrated samples may suggest increased Li^+^ ion conductivity, as well as perhaps an increase in proton mobility, through the SEI. However, the increasing thickness, and perhaps density, may also inhibit the transfer of reactants to the catalytically active surface.^52^ It may be that the combination of these factors contributes to the change in faradaic efficiency with increasing LiClO_4_ concentration.

Looking beyond lithium, our study also shows how sensitive the Li-mediated system is to even small perturbations in the LiClO_4_ concentration. Indeed, as discussed in ESI section 10,† we had many difficulties with very small amounts of LiClO_4_ contamination which delayed experiments by nearly a year. Had we not known from the work of Andersen *et al.*^12^ that the Tsuneto electrolyte could reproducibly make ammonia, we would have discounted LiClO_4_ altogether. Such findings echo those of Lazouski *et al.*^3^ who noted a very sharp peak in optimum ethanol concentration, as well as an optimum purity level for the LiBF_4_ salt. The community should carefully consider the sensitivity of the system before discounting materials for nitrogen reduction, which may either be contaminated or just slightly outside of the ammonia formation window with the right electrolyte stability, ion transport, nitrogen transport, and chemical potential of protons.

## 5. Conclusions and outlook

Until now, most research in the field has followed a more Edisonian approach of improving ammonia production without in-depth investigation of what exactly affects the performance. In this work, we have directly linked three crucial parameters for the Li-mediated system; the bulk electrolyte properties, the SEI content and the faradaic efficiency: we show that a moderate increase in faradaic efficiency is observed at 0.6 M LiClO_4_, whereas use of lower or higher concentrations limits performance. Fundamentally, this study of the LiClO_4_ system reveals that SEI composition is critical for stability, and that impaired SEI functionality results in decreased faradaic efficiency.

We discuss that the solvation environment of the Li^+^ ion has a significant impact on the structure of the SEI and the stability of the LiClO_4_ system. Increasing the Li salt concentration increases the likelihood of finding the salt anion within the Li^+^ ion solvation shell, which in turn increases the proportion of inorganic species in the SEI resulting from salt decomposition. The increased inorganic content of the SEI results in greater electrochemical stability, but this benefit must be balanced with the lower solubility of N_2_ gas in the more concentrated electrolyte and increased Li^+^ ion conductivity in the SEI. The problem of N_2_ solubility could be mitigated by operating at higher nitrogen partial pressure, or perhaps by using novel locally concentrated electrolytes.^53^

Importantly, this work represents an interesting battery-science motivated step forward in the optimisation of the lithium-mediated nitrogen reduction system. We have shown that it is possible to favourably tailor the SEI, even using a salt and organic solvent not typically used to promote good SEI formation in LiB science. Other avenues could be the use of battery additives to further tune SEI properties,^34^ or even drawing inspiration from the hydrophobic, anhydrous environment surrounding the catalytically active centre of nitrogenase^54,55^ to design an artificial SEI. Such a layer could allow for the field to move away from the requirement for *in situ* lithium plating which fixes the energy efficiency of the lithium-mediated nitrogen reduction at unfeasibly high values^22^ and instead rely on an active but much less scarce and expensive transition metal catalyst, such as those proposed by Skúlason *et al.*^10^ Such artificial SEI layers have been proposed in lithium-metal^56^ and lithium-sulfur batteries.^57^ It even opens the possibility for the use of an aqueous bulk electrolyte. Researchers have proposed that a stable, mostly inorganic SEI could provide improved kinetic stability, even in an aqueous battery.^34^ While such technologies require more investigation before they can be viable,^58^ the ability to perform aqueous nitrogen reduction in a much milder potential environment would be revolutionary.

## Author contributions

Conceptualisation: O. W., M. S., I. E. L. S., A. G., data curation: O. W., M. S., data analysis: O. W., M. S., Z. S., A. R., investigation: O. W., M. S., H. Y., S. F., Z. S., A. B., methodology – ammonia quantification: O. W., M. S., R. T., methodology – equipment design: O. W., methodology – visualisation: O. W., M. S., supervision: M. T., A. A., M. R., R. J., A. G., I. E. L. S., writing – original draft: O. W., M. S, writing – review: I. E. L. S., A. G., A. R., A. A., R. J., M. T., M. T., R. T., S. F., H. Y., Z. S., A. B., writing – editing and preparation of final manuscript: O. W.

## Conflicts of interest

The authors declare no conflicts of interest.

## Supplementary Material

TA-011-D2TA07686A-s001

## References

[cit1] Morlanés N., Katikaneni S. P., Paglieri S. N. (2021). *et al.*, A technological roadmap to the ammonia energy economy: Current state and missing technologies. Chem. Eng. J..

[cit2] Seh Z. W., Kibsgaard J., Dickens C. F. (2017). *et al.*, Combining theory and experiment in electrocatalysis: Insights into materials design. Science.

[cit3] Lazouski N., Schiffer Z. J., Williams K. (2019). *et al.*, Understanding Continuous Lithium-Mediated Electrochemical Nitrogen Reduction. Joule.

[cit4] Wang M., Kahn M. A., Mohsin I. (2021). *et al.*, Can sustainable ammonia synthesis pathways compete with fossil-fuel based Haber–Bosch processes?. Energy Environ. Sci..

[cit5] Comer B. M., Fuentes P., Dimkpa C. O. (2019). *et al.*, Prospects and Challenges for Solar Fertilizers. Joule.

[cit6] MacFarlane D. R., Cherepanov P. v., Choi J. (2020). *et al.*, A Roadmap to the Ammonia Economy. Joule.

[cit7] Chen J. G., Crooks R. M., Seefeldt L. C. (2018). *et al.*, Beyond fossil fuel–driven nitrogen transformations. Science.

[cit8] Smith C., Hill A. K., Torrente-Murciano L. (2020). Current and future role of Haber-Bosch ammonia in a carbon-free energy landscape. Energy Environ. Sci..

[cit9] Choi J., Suryanto B. H. R., Wang D. (2020). *et al.*, Identification and elimination of false positives in electrochemical nitrogen reduction studies. Nat. Commun..

[cit10] Skúlason E., Bligaard T., Gudmundsdóttir S. (2012). *et al.*, A theoretical evaluation of possible transition metal electro-catalysts for N_2_ reduction. Phys. Chem. Chem. Phys..

[cit11] Montoya J. H., Tsai C., Vojvodic A. (2015). *et al.*, The challenge of electrochemical ammonia synthesis: A new perspective on the role of nitrogen scaling relations. ChemSusChem.

[cit12] Andersen S. Z., Čolić V., Yang S. (2019). *et al.*, A rigorous electrochemical ammonia synthesis protocol with quantitative isotope measurements. Nature.

[cit13] Tsuneto A., Kudo A., Sakata T. (1994). Lithium-mediated electrochemical reduction of high pressure N_2_ to NH_3_. J. Electroanal. Chem..

[cit14] Tsuneto A., Kudo A., Sakata T. (1993). Efficient Electrochemical Reduction of N_2_ to NH_3_ Catalyzed by Lithium. Chem. Lett..

[cit15] Lazouski N., Chung M., Williams K. (2020). *et al.*, Non-aqueous gas diffusion electrodes for rapid ammonia synthesis from nitrogen and water-splitting-derived hydrogen. Nat. Catal..

[cit16] Cherepanov P. v., Krebsz M., Hodgetts R. Y. (2021). , *et al*., Understanding the Factors Determining the Faradaic Efficiency and Rate of the Lithium Redox-Mediated N_2_ Reduction to Ammonia. J. Phys. Chem. C.

[cit17] Du H. L., Chatti M., Hodgetts R. Y. (2022). *et al*., Electroreduction of nitrogen at almost 100% current-to-ammonia efficiency. Nature.

[cit18] Li S., Zhou Y., Li K. (2022). *et al*., Electrosynthesis of ammonia with high selectivity and high rates via engineering of the solid-electrolyte interphase. Joule.

[cit19] Suryanto B. H. R., Matuszek K., Choi J. (2021). *et al.*, Nitrogen reduction to ammonia at high efficiency and rates based on a phosphonium proton shuttle. Science.

[cit20] Andersen S. Z., Statt M. J., Bukas V. J. (2020). *et al.*, Increasing stability, efficiency, and fundamental understanding of lithium-mediated electrochemical nitrogen reduction. Energy Environ. Sci..

[cit21] Li K., Andersen S. Z., Statt M. J. (2021). *et al.*, Enhancement of lithium-mediated ammonia synthesis by addition of oxygen. Science.

[cit22] Westhead O., Jervis R., Stephens I. E. L. (2021). Is lithium the key for nitrogen electroreduction?. Science.

[cit23] Bagger A., Wan H., Stephens I. E. L. (2021). *et al.*, Role of Catalyst in Controlling N_2_ Reduction Selectivity: A Unified View of Nitrogenase and Solid Electrodes. ACS Catal..

[cit24] Varley J. B., Wang Y., Chan K. (2015). *et al.*, Mechanistic insights into nitrogen fixation by nitrogenase enzymes. Phys. Chem. Chem. Phys..

[cit25] Bukas V. J. , Nørskov J. K., A Molecular-Level Mechanism of the Biological N_2_ Fixation A molecular-level mechanism of the biological N_2_ fixation, ChemRxiv, 2019, preprint, 10.26434/chemrxiv.10029224.v1

[cit26] Yandulov D. v., Schrock R. R. (2003). Catalytic Reduction of Dinitrogen to Ammonia at a Single Molybdenum Centre. Science.

[cit27] Chalkley M. J., Drover M. W., Peters J. C. (2020). Catalytic N_2_-to-NH_3_ (or -N_2_H_4_) Conversion by Well-Defined Molecular Coordination Complexes. Chem. Rev..

[cit28] Peled E., Menkin S. (2017). Review—SEI: Past, Present and Future. J. Electrochem. Soc..

[cit29] Schwalbe J. A., Statt M. J., Chosy C. (2020). *et al.*, A Combined Theory-Experiment Analysis of the Surface Species in Lithium-Mediated NH_3_ Electrosynthesis. ChemElectroChem.

[cit30] Singh A. R., Rohr B. A., Statt M. J. (2019). *et al.*, Strategies toward Selective Electrochemical Ammonia Synthesis. ACS Catal..

[cit31] Westhead O., Spry M., Bagger A., Shen Z., Yadegari H., Favero S., Tort R., Titirici M., Ryan M. P., Jervis R., Katayama Y., Aguadero A., Regoutz A., Grimaud A., Stephens I. E. L. (2022). DFT data for “The Role of Ion Solvation in Lithium Mediated Nitrogen Reduction” [Data set]. Zenodo.

[cit32] Yao Y., Chen X., Yan C. (2021). *et al.*, Regulating Interfacial Chemistry in Lithium-Ion Batteries by a Weakly Solvating Electrolyte. Angew. Chem..

[cit33] Yamada Y., Wang J., Ko S. (2019). *et al.*, Advances and issues in developing salt-concentrated battery electrolytes. Nat. Energy.

[cit34] Zhang Z., Smith K., Jervis R. (2020). *et al.*, Operando Electrochemical Atomic Force Microscopy of Solid–Electrolyte Interphase Formation on Graphite Anodes: The Evolution of SEI Morphology and Mechanical Properties. ACS Appl. Mater. Interfaces.

[cit35] Suo L., Borodin O., Gao T. (2015). *et al.*, ‘Water-in-salt’ electrolyte enables high-voltage aqueous lithium-ion chemistries. Science.

[cit36] Li K., Shapel S. G., Hoch D. (2022). *et al.*, Increasing Current Density of Li-Mediated Ammonia Synthesis with High Surface Area Copper Electrodes. ACS Energy Lett..

[cit37] Krempl K., Pedersen J. B., Kibsgaard J. (2022). *et al.*, Electrolyte acidification from anode reactions during lithium mediated ammonia synthesis. Electrochem. Commun..

[cit38] Sažinas R., Andersen S. Z., Li K. (2021). *et al.*, Towards understanding of electrolyte degradation in lithium-mediated non-aqueous electrochemical ammonia synthesis with gas chromatography-mass spectrometry. RSC Adv..

[cit39] Peljo P., Girault H. H. (2018). Electrochemical potential window of battery electrolytes: The HOMO-LUMO misconception. Energy Environ. Sci..

[cit40] Schürmann A., Haas R., Murat M. (2018). *et al.*, Diffusivity and Solubility of Oxygen in Solvents for Metal/Oxygen Batteries: A Combined
Theoretical and Experimental Study. J. Electrochem. Soc..

[cit41] Das D. (2008). Ion Association and Solvation Behavior of Some Lithium Salts in Tetrahydrofuran. A Conductivity and Raman. J. Solution Chem..

[cit42] Kameda Y., Ebina S., Amo Y., Usuki T., Otomo T. (2016). Microscopic Structure of Contact Ion Pairs in Concentrated LiCl^−^ and LiClO_4_ -Tetrahydrofuran Solutions Studied by Low-Frequency Isotropic Raman Scattering and Neutron Diffraction with ^6^Li/^7^Li Isotopic Substitution Methods. J. Phys. Chem. B.

[cit43] Daubert J. S., Afroz T., Borodin O. (2022). *et al.*, Solvate Structures and Computational/Spectroscopic Characterization of LiClO_4_ Electrolytes. J. Phys. Chem. C.

[cit44] Chabanel M., Legoff D., Touaj K. (1996). Aggregation of perchlorates in aprotic donor solvents Part 1.-Lithium and sodium perchlorates. J. Chem. Soc., Faraday Trans..

[cit45] Blair S. J., Doucet M., Browning J. F. (2022). *et al.*, Lithium-Mediated Electrochemical Nitrogen Reduction: Tracking Electrode–Electrolyte Interfaces via Time-Resolved Neutron Reflectometry. ACS Energy Lett..

[cit46] Elder S. H., DiSalvo F. J., Topor L. (1993). Thermodynamics of ternary nitride formation by ammonolysis: application to lithium molybdenum nitride (LiMoN_2_), sodium tungsten nitride (Na_3_WN_3_), and sodium tungsten oxide nitride (Na_3_WO_3_N). Chem. Mater..

[cit47] Standard thermodynamic properties of chemical substances, in CRC Handbook of Chemistry and Physics, ed. Rumble J. R., CRC Press/Taylor & Francis, Boca Raton, FL, 2020

[cit48] Guo L., Thornton D. B., Koronfel M. A. (2021). *et al.*, Degradation in lithium ion battery current collectors. J. Phys.: Energy.

[cit49] Dubouis N., Marchandier T., Rousse G. (2021). *et al.*, Extending insertion electrochemistry to soluble layered halides with superconcentrated electrolytes. Nat. Mater..

[cit50] Wang E., Dey S., Liu T. (2020). *et al.*, Effects of Atmospheric Gases on Li Metal Cyclability and Solid-Electrolyte Interphase Formation. ACS Energy Lett..

[cit51] Chen Y. C., Ouyang C. Y., Song L. J. (2011). *et al.*, Electrical and lithium ion dynamics in three main components of solid electrolyte interphase from density functional theory study. J. Phys. Chem. C.

[cit52] Lazouski N., Steinberg K. J., Gala M. L. (2022). *et al.*, Proton Donors Induce a Differential Transport Effect for Selectivity toward Ammonia in Lithium-Mediated Nitrogen Reduction. ACS Catal..

[cit53] Zheng Y., Fernando S. A., Ponce V., Seminario J. M., Co X., Zhang J., Balbuena P. B. (2019). Localized high concentration electrolyte behavior near a lithium–metal anode surface. J. Mater. Chem. A.

[cit54] Durrant M. C. (2001). Controlled protonation of iron–molybdenum cofactor by nitrogenase : a structural and theoretical analysis. Biochem. J..

[cit55] Dance I. (2012). The controlled relay of multiple protons required at the active site of nitrogenase. Dalton Trans..

[cit56] Fan L., Guo Z., Zhang Y., Wu X., Zhao C., Sun X., Yang G., Feng Y., Zhang N. (2020). Stable artificial solid electrolyte interphase films for lithium metal anode *via* metal–organic frameworkscemented by polyvinyl alcohol. J. Mater. Chem. A.

[cit57] Ma G., Wen Z., Wang Q., Shen C., Jin J., Wu X. (2014). Enhanced cycle performance of a Li–S battery based on a protected lithium anode. J. Mater. Chem. A.

[cit58] Droguet L., Hobold G. M., Lagadec M. F. (2021). *et al.*, Can an Inorganic Coating Serve as Stable SEI for Aqueous Superconcentrated Electrolytes?. ACS Energy Lett..

