# Correction: The role of ion solvation in lithium mediated nitrogen reduction

**DOI:** 10.1039/d3ta90009f

**Published:** 2023-01-13

**Authors:** O. Westhead, M. Spry, A. Bagger, Z. Shen, H. Yadegari, S. Favero, R. Tort, M. Titirici, M. P. Ryan, R. Jervis, Y. Katayama, A. Aguadero, A. Regoutz, A. Grimaud, I. E. L. Stephens

**Affiliations:** a Department of Materials, Imperial College London UK i.stephens@imperial.ac.uk; b Solid-State Chemistry and Energy Laboratory, UMR8260, CNRS, Collège de France France alexis.grimaud@bc.edu; c Department of Chemistry, University of Copenhagen Denmark; d Department of Chemical Engineering, Imperial College London UK; e The Faraday Institution, Quad One, Harwell Science and Innovation Campus Didcot OX11 0RA UK; f Electrochemical Innovation Lab, Department of Chemical Engineering, University College London UK; g SANKEN, Osaka University Japan; h Instituto de Ciencia de Materiales de Madrid ICMM-CSIC Spain; i Department of Chemistry, University College London UK; j Réseau sur le Stockage Electrochimique de l'Energie (RS2E), CNRS FR 3459 80039 Amiens Cedex 1 France; k Department of Chemistry, Merkert Chemistry Center, Boston College Chestnut Hill MA USA

## Abstract

Correction for ‘The role of ion solvation in lithium mediated nitrogen reduction’ by O. Westhead *et al.*, *J. Mater. Chem. A*, 2023, https://doi.org/10.1039/D2TA07686A.

The authors regret an error in their calculation of the yield rate in Fig. 1b. Due to an error with the unit conversion the peak yield rate at 0.6 M LiClO_4_ was incorrectly given as 60 ± 3 nmol cm^−2^ s^−1^ (*n* = 3). The corrected yield rate is 0.53 ± 0.04 nmol cm^−2^ s^−1^ (*n* = 3) and the corrected version of Fig. 1b is provided herein.

**Fig. 1:**
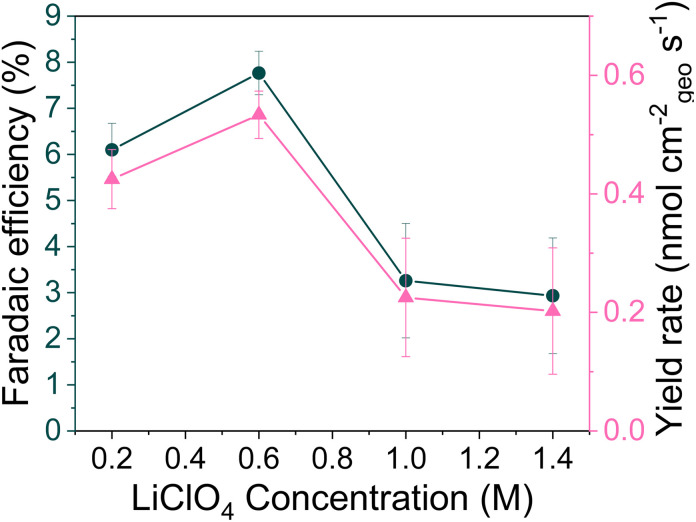
(b) The change in faradaic efficiency and yield rate with LiClO_4_ concentration (*n* = 3 separate experiments, error bar is standard error in the mean) for a chronopotentiometry experiment at an applied constant current of −2 mA cm^−2^ until −10C is passed.

An independent expert reviewed the data provided by the authors and concluded that it does not change the discussion or conclusions presented in the article.

The Royal Society of Chemistry apologises for these errors and any consequent inconvenience to authors and readers.

## Supplementary Material

