# Supplementary material for: The role of ion solvation in lithium mediated nitrogen reduction
Source: J Mater Chem A Mater. 2022 Nov 28;11(24):12746–58. doi: 10.1039/d2ta07686a (PMC10281334; doi:10.1039/d2ta07686a)
Supplement: TA-011-D2TA07686A-s001 [file TA-011-D2TA07686A-s001.pdf]

Electronic Supplementary Material (ESI) for Journal of Materials Chemistry A.
This journal is © The Royal Society of Chemistry 2022

The Role of Ion Solvation in Lithium Mediated Nitrogen Reduction: Supplementary Information

O. Westhead[1,2], M. Spry[1], A. Bagger[3,4], Z. Shen[1], H. Yadegari[1], S. Favero[4], R. Tort[4], M. Titirici[4,5], M.P. Ryan[1,5], R. Jervis[5,6], Y. Katayama[7], A. Aguadero[1,5,8], A. Regoutz[9], A. Grimaud[2,10,11]*, I. E. L Stephens[1,5]*

[1]Department of Materials, Imperial College London

[2] Solid-State Chemistry and Energy Laboratory, UMR8260, CNRS, Collège de France

[3]Department of Chemistry, University of Copenhagen

[4]Department of Chemical Engineering, Imperial College London

[5]The Faraday Institution, Quad One, Harwell Science and Innovation Campus, Didcot, OX11 0RA, UK

[6]Eletrochemical Innovation Lab, Department of Chemical Engineering, University College London

[7]SANKEN, Osaka University

[8]Instituto de Ciencia de Materiales de Madrid ICMM-CSIC

[9]Department of Chemistry, University College London

[10]Réseau sur le Stockage Electrochimique de l'Energie (RS2E), CNRS FR 3459, 80039 Amiens Cedex 1, France

[11]Department of Chemistry, Merkert Chemistry Center, Boston College, Chestnut Hill, MA

1 – Materials

2 – Electrochemical cell preparation

3 – Electrochemical testing

4 – Characterisation sample preparation

5 – Ammonia Quantification

6 – Contamination testing

7 – Raman Spectroscopy

8 – Solubility and Diffusivity measurements

9 – DFT calculations

10 – A note on chemical contamination

1. Materials

Tetrahydrofuran (anhydrous, ≥99.9%, inhibitor-free), lithium perchlorate (99.99%, battery grade) sodium salicylate ($C_6H_4(COONa)(OH)$, ReagentPlus®, ≥99.5%) and sodium hydroxide solution (30%, Suprapur) were purchased from Sigma-Aldrich. Anhydrous lithium perchlorate (99%, anhydrous) and sodium pentacyanonitrosylferrate(III) dihydrate (ACS, 99 – 102 %) were purchased from Alfa Aesar. Sodium hypochlorite (14% Cl_2 in aqueous solution, GPR RECTAPUR) and sodium hydroxide (pellets AnalaR NORMAPUR) were purchased from VWR. Ethanol (99.9%, Extra Dry, AcroSeal) was purchased from Fisher Scientific. Platinum mesh (wire diameter 0.1mm, nominal aperture 0.4 mm, purity 99.9%), Platinum wire (diameter 0.5 mm, 99.99%, as drawn), Copper wire (0.5 mm, 99.99%, as drawn), molybdenum foil (0.125 mm thick, annealed, 99.9%) and copper foil (0.125 mm thick, as rolled,

99.99%) were purchased from Goodfellow. Single compartment glass cell was custom made by Artistic and Scientific Glassware, Oxford. Purifiers for the Ar and N_2 gas lines providing purity levels of H_2O, H_2, CO_2, O_2, CO, nonmethane hydrocarbon (NMHC), CH_4, NH_3, NO_x to < 0.5 ppb were purchased from NuPure. N6 Ar and N6 N2 gas was purchased from BOC. Electrochemistry and electrolyte preparation was carried out in an Ar atmosphere glovebox (MBraun, H_2O <0.3 ppm, O_2 < 0.3 ppm).

The lithium perchlorate supplied by Alfa-Aesar was used for all the experiments in the main body of this paper.

2. Electrochemical cell preparation

$LiClO_4$, THF and ethanol were used to make electrolytes of 99:1 vol% THF:EtOH and various $LiClO_4$ molar concentrations. The THF and ethanol were used as purchased. The $LiClO_4$ was dried under vacuum at 100°C for at least 12 hours. The typical electrolyte water content was 50 – 70 ppm measured by Karl Fisher Titration, as shown in Table S2.

Either Mo or Cu 1 cm^2 working electrodes were used. Mo electrodes were used for electrochemical testing, and Cu electrodes were used for SEI characterisation to avoid the overlap of the Mo 3p and N 1s core levels in XPS. Cu wire was used as a current collector. Both electrodes were dipped in 4M HCl and rinsed with EtOH prior to electrochemical measurements. Mo electrodes were polished with 400, 1500 and 2500 grit silicon carbide paper to a mirror finish and then sonicated in ethanol. The Pt mesh counter electrode and Pt wire pseudo-reference electrode were flame annealed prior to use. The single-compartment glass cell was then assembled such that the working and counter electrode were approximately 1 cm apart with the Pt wire pseudo-reference in between. The cell was then taken into the glovebox and filled with between 11 ml and 15 ml of electrolyte. A sample of blank electrolyte was taken for ammonia quantification. The cell was then connected into a closed gas line shown in figure S1. Ar gas was passed through the cell to leak test. For nitrogen reduction experiments, the cell was pre-saturated with N_2 gas at a flow rate of 20 ml/min for 30 minutes. Electrochemistry was carried out at around 5 ml/min. After electrochemistry is finished, the cell is purged with Ar at a minimum of 20 ml/min for around 30 minutes to avoid contaminating the glovebox atmosphere with N_2. Both Ar and N_2 gas were passed through separate purifiers for inerts upstream of the experiment. A PTFE coated magnetic stirrer was used to agitate the electrolyte.

After electrochemistry, the cell was disassembled inside the glovebox. The electrolyte was sampled for ammonia quantification. The glass cell, rubber stoppers, magnetic stirrer, Pt wire pseudo-reference and Pt mesh counter electrode were removed from the glovebox and boiled in ultra-pure water (>18.2 MΩ, Sartorius) for one hour. The working electrode was either kept inside the Ar glovebox for later characterisation or was removed from the glovebox and cleaned in 4 M HCl to remove any SEI species. The glass cell, stoppers, Pt wire pseudo-reference and Pt mesh counter electrode were stored in a glass drying oven at 70°C. The working electrode was stored in air.

3. Electrochemical testing

All experiments were carried out at ambient temperature and pressure.

The cell was allowed to rest at open circuit voltage (OCV) for 30s to ensure a stable OCV. An impedance spectrum was taken to determine the uncompensated resistance; we used this initial value to correct the potential during the electrocatalytic measurements. The impedance of the counter electrode is also taken during this measurement and the uncompensated resistance used to correct the potential of the counter electrode. A linear sweep voltammogram (LSV) was taken until lithium plating is clearly seen. All potentials are referenced to the potential value at which this occurs. A constant current

density of -2 mA cm^{-2} is then applied until -10 C of charge is passed (chronopotentiometry, CP). A second impedance spectrum is taken at OCV, which is now the lithium plating potential, to determine the SEI impedance. For the PEIS spectra, data points were collected between 200kHz and 100mHz about OCV at an amplitude of 10 mV. See figure S2 for example plots. Ohmic drop data can be found in Table S1 and generally decreased with increasing salt concentration. The ohmic drop did not change significantly between the initial and final PEIS spectra. The first ohmic drop measurement was used to correct the electrochemical data.

4. Characterisation sample preparation

Copper electrodes kept for characterisation were cut in half inside the Ar glovebox. One half was characterised by XPS and the other by ToF-SIMS.

a. XPS sample preparation & characterisation

Since XPS is very surface sensitive, the XPS samples were rinsed in 0.1 ml THF to remove any dried electrolyte on the surface. While this may have removed some weakly bound species, it avoids results being confused with electrolyte signals. The samples were then loaded into a glove box transfer module and affixed using a Cu clip. The samples were then transferred under exclusion of air to the XPS system (ThermoFisher Scientific K-Alpha+, monochromated, microfocused Al Kα X-ray source, 400 µm spot size). Base pressure was 2 x 10^{-9}. The flood gun was used for charge compensation. Survey spectra were taken for all three samples, along with Cl 2p, O 1s and Li 1s core levels. No N 1s features were observed.

Peak fitting was performed using Thermo Scientific™ Avantage™ software. The 'smart' background was used. Peak widths were allowed to vary between constraints of 0.5 and at least 2 eV. The Lorentzian-Gaussian mix was allowed to vary between 10 and 40 %.

b. ToF-SIMS sample preparation & characterisation

ToF-SIMS samples were heat sealed in moisture barrier bags (RS Components, United Kingdom) and transported to a different Ar-glovebox (H_2O< 0.6 ppm, O_2 < 0.6 ppm) where they were mounted on a back-mount sample holder and loaded into a vacuum-transfer suitcase. The samples were then transferred to the ToF-SIMS machine (TOF.SIMS5 IONTOF GmbH, Münster, Germany) under vacuum. The vacuum transfer suitcase was opened until the pressure of the loadlock chamber was lower than 3x10^{-5} mbar. The analysis was performed with a 25keV Bi^+ primary ion beam with the current of 1.2 pA and the high current bunched mode was applied in order to achieve high mass resolution. Samples were sputtered using GCIB (Gas Cluster Ion Beam) Ar_n^+(n=1159) at 10 nA, which is very gentle and minimises sample damage[1]. Time limitations meant that only the negative spectrum was collected, which generally has a higher yield compared to positive for oxides and chlorides[2,3]. Crater depth was measured after SIMS analysis with a Zygo NewView 200 3D optical white light interferometer (height resolution ~ 1nm).

5. Ammonia Quantification

The salicylate method described below was adapted slightly from that described by Lazouski et al[4].

Sodium nitroprusside solution: 149 mg of sodium pentacyanonitrosylferrate(III) dihydrate was dissolved in 10 ml ultra-pure water to make a 0.05 M solution. The solution was stored at 4°C in the dark.

Sodium salicylate purification: 40g of sodium salicylate was dissolved in 300 ml ultra-pure water. 50 ml of 6 M HCl was added dropwise to form a white precipitate (salicylic acid), which was removed by

filtration and washed with ultra-pure water. The salicylic acid was dried at 40°C under vacuum overnight.

Salicylate solution: For every 10g of dry salicylaic acid, 17.5 ml of 4M NaOH (suprapur) and 290 µl sodium nitroprusside solution was added. The solution was diluted to 29 ml and contained 2.5 M sodium salicylate and $5x10^{-4}$ M sodium nitroprusside. The solution was stored at 4°C in the dark.

Alkaline solution: 800 mg of sodium hydroxide (VWR pellets) was dissolved in 50 ml ultra-pure water to obtain 0.4 M NaOH. The solution was stored at 4°C in the dark, along with the sodium hypochlorite solution. Just before quantification, NaOH was mixed with the stock sodium hypochlorite solution in a 9:1 ratio to obtain approximately 1% sodium hypochlorite.

Sample preparation: Immediately after the end of an electrochemistry experiment, 4 samples of 400 µl of the electrolyte were collected and removed from the glovebox along with 800 µl of blank electrolyte. For every 400 µl of sample, 20 µl of 4 M HCl was added. The samples were then evaporated in a water bath between 65 and 70 °C until only a dry residue was obtained. For the overnight experiments, a smaller quantity of the electrolyte was used for quantification.

Blank samples: The samples were re-dissolved in ultra-pure water to obtain 2 x 2 ml samples. These were transferred to cuvettes. 580 µl of ultra-pure water was added to one sample. 290 µl of the salicylate solution followed by 290 µl of the alkaline solution was added to the other. The samples were left for 45 mins to develop in the dark.

Used electrolyte samples: The samples were re-dissolved in ultra-pure water to obtain 4 x 2 ml samples, which were transferred to cuvettes. 290 µl of the salicylate solution was added followed by 290 µl of the alkaline solution. The samples were left for exactly 45 mins to develop in the dark.

UV-vis spectroscopy: The absorbance of the samples was measured by UV-vis spectroscopy between 400 nm and 900 nm. The absorbance peak at approximately 650 nm was corrected with the absorbance at the trough (at 900nm) to account for any discolouration of the THF. The calibration curves in figure S2 were used to calculate the concentration of ammonia in the electrolyte.

Calibration curves: NH_4Cl salt was dissolved in ethanol to yield solutions of 1000 ppm and 5000ppm. Electrolytes of 0.2 M, 0.6 M, 1 M and 1.4 M $LiClO_4$ were made using the NH_4Cl containing ethanol to give a certain concentration of NH_4Cl in the electrolyte. These were then diluted with blank electrolyte to yield a range of different concentrations of NH_4Cl. 4 x 400ul samples were taken of each concentration of NH_4Cl containing electrolyte and treated in the same way as described above. Calibration curves (figure S2 a - d) were then plotted. There is a strong reaction between the $LiClO_4$ salt and the salicylate reagents, as investigated by Shao-Horn and coworkers[5]. The 0.2 M and 0.6 M calibration curves were linear, with R^2 values of 0.98654 and 0.98615 respectively. The 1 M and 1.4 M calibration curves were not linear and are approximated by a cubic relationship. The R^2 values for these curves are 0.99302 and 0.98477 respectively. Due to the very small absorbance for the 1M and 1.4M samples below 10 ppm NH_4Cl, it is difficult to accurately quantify the ammonia content of these samples. However, we can show that the general trend is a drop off in Faradaic efficiency after 0.6 M $LiClO_4$. For the overnight experiments, 100ul samples were used due to the higher concentrations of ammonia in the electrolyte. For this calibration curve, it was difficult to obtain the higher concentrations of ammonia in the sample by the aforementioned method. In this case, for concentrations up to 50 ppm, samples were prepared in the same way as for the other curves. For concentrations above 50ppm, a few microlitres of 5000 ppm NH_4Cl ethanol solution was added to blank ammonia solutions to yield higher concentrations. A linear relationship was obtained with an R^2 value of 0.98607. Figure S3 shows the calibration curves obtained.

Faradaic efficiency calculation: The Faradaic efficiency was calculated using the below equation,

$$FE_{NH3}\,(\%) = \frac{3\,F\,C_{NH3}V}{Q},$$

where F is the Faraday constant, C_{NH3} is the concentration of ammonia as measured by the salicylate method, V is the volume of electrolyte as measured at the end of the experiment and Q is the charge passed.

6. Contamination testing

To ensure the validity of our results, both Ar and N_2 blanks were carried out as per the protocol laid out by Andersen et al[6]. Since the system has already been rigorously verified by the aforementioned authors, isotopically labelled experiments were not carried out. For an Ar blank, the same electrochemical procedure was used as for a nitrogen reduction experiment, except using Ar as the feed gas. This test detects contaminants present in the cell. For the N_2 blank, N_2 gas was passed through the cell at open circuit potential for the same amount of time as a normal electrochemistry experiment. The 0.2 M $LiClO_4$ and 0.6 M $LiClO_4$ electrolytes were tested, the first to compare our results to those reported by Andersen et al.[6], and the second to confirm that the peak in Faradaic efficiency was valid. The tests were also carried out overnight to validate the longer-term experiments. No ammonia was detected in any condition, as shown in Table S1.

7. Raman spectroscopy

Samples of THF with varying molar concentrations of $LiClO_4$ were prepared inside an Ar glovebox. Glass capillaries were sealed at one end using a blowtorch. Samples were injected into the capillaries using a syringe and needle. The other end of the capillary was sealed using parafilm. Samples were then transferred to the Raman spectrometer.

Raman spectra were collected using an inVia Renishaw confocal Raman microscope operated with an incident laser beam at 532 nm focused through a 50x objective (Leica). The laser intensity was set to 25 mW and Raman spectra were collected under extended mode between 150-3500 cm^{-1} wavenumber with the exposure time of 10 s.

8. Solubility and Diffusivity measurements

Samples of THF with varying molar concentrations of $LiClO_4$ were prepared inside an Ar glovebox. A porosity analyser 3Flex from Micromeritics was used to measure N_2 solubility and diffusivity following the below method, similar to that used by Zubeir et al[7].

Freeze-thaw: To remove any dissolved gas in the solvent, we performed 3 freeze-thaw cycles in-situ.

2ml was used for each measurement. After loading the sample, the tube was secured on the porosity analyser. The sample was then frozen by immersion in a liquid nitrogen bath. The manifold line was put under vacuum and the sample valves were opened, to degas the solidified samples. Vacuum was applied for 10 minutes, at the end of which, the sample valves were closed again and the nitrogen bath removed. The samples were then left to thaw under vacuum, during this step gas bubbles were visible. The process was repeated until no more bubbles were observed upon thawing (usually 3 times).

Free volume determination: The free volume is an essential parameter to calculate nitrogen solubility from the pressure vs time data. In normal porosity analysis with solid samples, the free volume is

determined by degassing, followed by dosing a non-adsorptive gas (usually Helium) to around 0.3bar.

This approach cannot be used for the case of low vapour pressure solvents. Therefore, the free volume was initially determined in absence of the sample and then the sample volume (2ml) was subtracted.

Solubility data points: After the freeze-thawing cycles, the sample was once again frozen with a liquid nitrogen bath. Nitrogen was fed into the system to reach a pressure of 0.2 bar. The sample valves were again closed, the nitrogen bath removed, and the pressure left to equilibrate, using an equilibration interval of 1 hour.

The initial pressure (0.2bar) was selected because around half of the sample tube is surrounded by liquid nitrogen, so upon removal of the nitrogen bath the pressure approximately doubles, obtaining a pressure above the saturation pressure of THF at 25°C (0.24 bar).

A known amount of nitrogen is dosed and the pressure recorded in steps of 0.2 bar up to 1 bar, which is the limit of the machine. The equilibration time for these measurements was 15 minutes and each data point took 2 to 5 hours to equilibrate. The pressure was then reduced to 0.4 bar again and the process repeated 3 times. The reported data represent the average of 3 runs (with the same sample).

To extrapolate nitrogen solubility, the mixture was assumed to obey Raoult's law.

Calculating N_2 solubility:

Short name	Description
V_{free}	Free volume (volume of the sample holder minus that of the sample), cm^3
V_{dosed}	Total gas volume dosed (cumulative, cm^3 stp)
T_{sample}	Temperature of the sample bath (K)
T_{stp}	Temperature at standard T,P (298K)
P_{meas}	Measured pressure in the tupe (mmHg)
P_{stp}	Pressure at standard T, P (750mmHg)
$V_{ads,stp}$	Adsorbed volume at standard T,P (cm^3 stp)
n_{ads}	Moles of adsorbed gas
P_{theor}	Theoretical pressure= pressure that we would expect in the tube if no gas was absorbed

$$n_{dosed} = \frac{V_{dosed} P_{stp}}{T_{stp} R}$$

$$P_{theor} = \frac{n_{dosed} R T_{sample}}{V_{free}}$$

$$\Delta P = P_{meas} - P_{theor}$$

$$n_{ads} = \frac{\Delta P V_{free}}{R T_{sample}} = \ldots = \frac{P_{meas} V_{free}}{R T_{stp}} - \frac{V_{dosed} P_{stp} T_{sample}}{R T_{stp}^2}$$

$$V_{ads,stp} = \frac{R T_{stp} n_{ads}}{P_{stp}} = \frac{V_{free} P_{meas}}{P_{stp}} - \frac{V_{dosed} T_{sample}}{T_{stp}}$$

Calculating N_2 diffusivity:

The diffusion coefficient is calculated by fitting the solution of the 1d Fick's equation

Fick's Second Law

$$\frac{\partial C}{\partial t} = D\frac{\partial^2 C}{\partial z^2}$$

Solution

$$\overline{C} = \frac{1}{L}\int_0^L C\,dt$$

$$\overline{C} = C_s\left(1 - 2\left(1 - \frac{C_o}{C_s}\right)\sum_{\lambda=0}^{\infty} \exp\left(\frac{\lambda_n D t}{L^2}\right)/\lambda_n\right), \quad \lambda_n = \left(n + \frac{1}{2}\right)^2\pi^2$$

By fitting the equation, we can calculate the steady state N_2 concentration and the diffusion coefficient. Notice that the solution is an infinite sum but has been approximated to the first 10. See figure S4 for details.

9. DFT calculations

Computational details:

The computational work is split into two. Part 1 which is related to running ab-initio molecular dynamics (AIMD) for different LiClO4 concentrations and Part 2 which was related to simulating Raman spectra of smaller molecular complexes. For both part 1 and part 2 the atomic structures were created using ASE[8].

The AIMD simulations was carried out at the generalized gradient approximation−density functional theory (GGA-DFT) level of theory, with the projector augmented wave method together with the PBE functional as implemented in the GPAW software[9]. The AIMD was run at a constant temperature of 300 K (using Berendsen NVT dynamics[10], with a time step of 0.5 fs and a time temperature cooling constant of 100 fs). To get sufficient sampling, only the gamma point is used together with a plane-wave cut-off at 350 eV and relatively low self-consistency criteria. These low setting are needed to enable sufficient sampling of low LiClO4 concentration, which corresponds to a large unit cell. The initial 0.5 M LiClO4 is built by using a LiClO4 in THF density of 1,121 g/cm^3. When higher concentration is simulated the unit cell shape (14.9 Å x 14.9 Å x 14.9 Å) is fixed and only the number of THF/Li/ClO4 molecules is changed. Hence, this methods of building the electrolyte models does not take care of changes in LiClO4/THF density with different concentrations and as the atomic models can only be created with integer addition and removal the models correspond to slight changes around the actual labelled concentration. However, trends for ion-pairing and bandgaps with increasing concentration is captured. The model systems are shown in Table S4.

The Raman spectra simulations was carried out using a two-step procedure as described in GPAW using the framework of M. Walter and M. Moseler[11]. First the vibrational frequencies are found using the Infrared module with the following computational settings; a 0.2 grid, the PBE functional and low forces of 0.01 eV/Å using the FIRE algorithm. Following the Raman excitations was calculated at each displacement using linear response TDDFT, as this is computationally consuming a coarser grid of 0.25 was used. When plotting frequency and intensities of the Raman data a broadening of 10 gamma was used.

Structures and scripts are available on Zenodo: https://doi.org/10.5281/zenodo.7378084

10. A note on chemical contamination

For a long time, we struggled with contamination in our system. We had initially been using $LiClO_4$ and indophenol reagents supplied by Sigma-Aldrich, as used by Andersen et al[6], but suddenly our experiments stopped producing ammonia. We believe the reason for this was contamination in both these chemicals. The alkaline hypochlorite solution supplied by Sigma-Aldrich contained too little hypochlorite to produce a blue enough solution to be consistent with our initial calibration curve, and the batch of $LiClO_4$ salt contained a higher than usual amount of magnesium according to the certificate of analysis. We suspect that this magnesium adversely affected something in the system. After discussions with PhD researchers and postdocs (mainly Katja Li, Dr Suzanne Z. Andersen and Dr Mattia Saccoccio) from the Technical University of Denmark we switched $LiClO_4$ suppliers to Alfa Aesar, which had a lower magnesium content, and began using the salicylate method and were able to produce ammonia again. We also tested a different batch of Sigma-Aldrich $LiClO_4$ and were able to replicate our original experiments. However, the salicylate method is not ideal for use with $LiClO_4$ containing electrolytes which has caused uncertainty in our ammonia quantification. Shao-Horn and coworkers' study on interferents with the salicylate method shows that $LiClO_4$ causes a shift in the peak position and a decrease in peak height[5], which makes it difficult to accurately measure small ammonia concentrations. It also caused our calibration curves to stray from linearity at higher $LiClO_4$ concentrations. For the purposes of this publication, where the focus is mainly on stability, this uncertainty is acceptable. However, for future investigations we will use a modified version of the indophenol blue method.

If we had not known that the system could produce ammonia, it would be natural to discount the $LiClO_4$ as a suitable salt candidate. This presents a significant challenge for the community; assuming such contamination problems are not unique, we may be discounting salts and solvents based on contamination rather than their actual ability to produce ammonia. We suggest that the community should test new salts and solvents from a few different batches and suppliers to ensure that they are not being deceived by contamination. Lazouski et al. already looked at different batches of $LiBF_4$ and found that only a certain product from Sigma-Aldrich gave reproducible results[4].

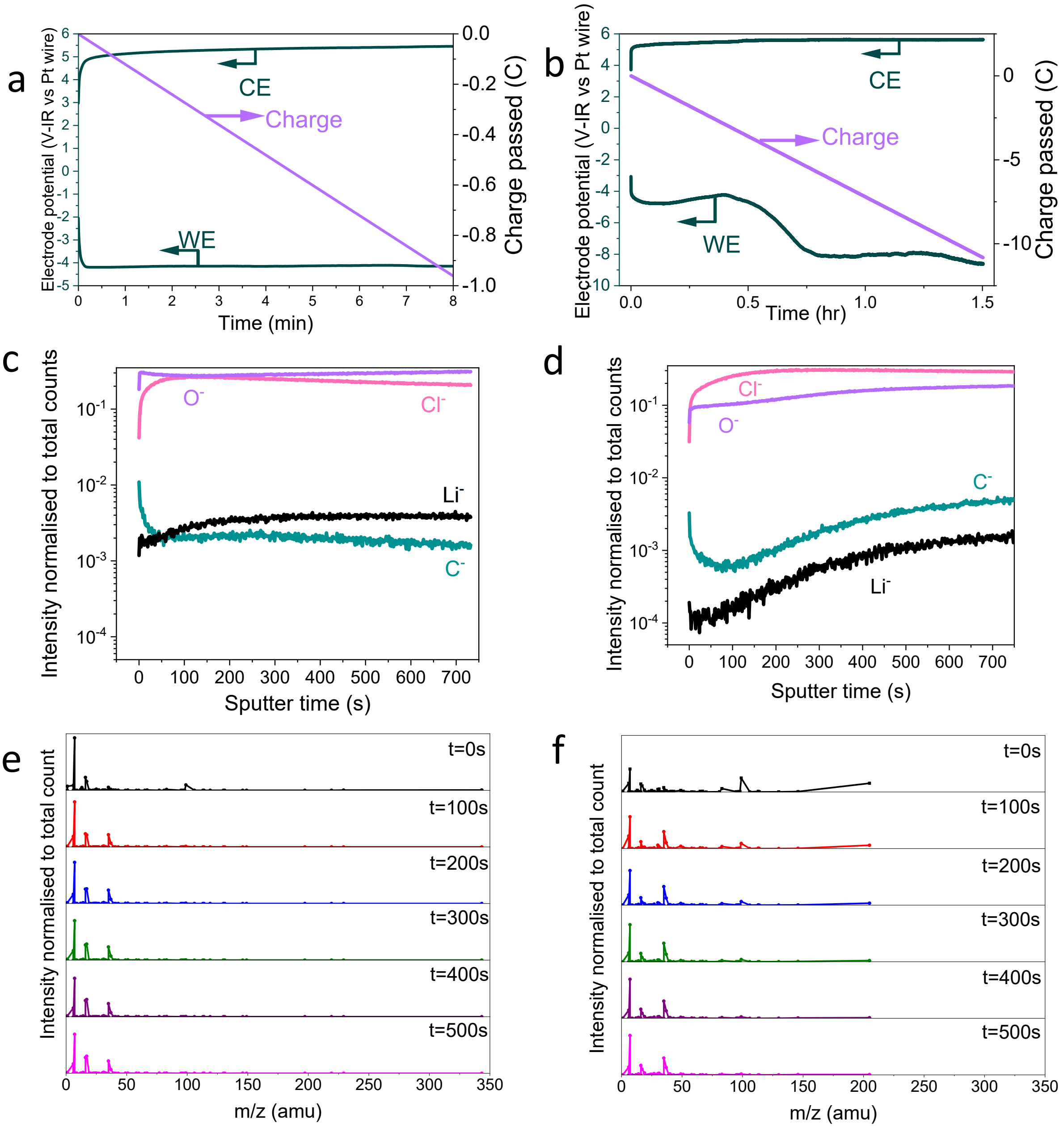

Figure S1 Initial ToF-SIMS results using 0.2 M $LiClO_4$ from Sigma-Aldrich in 99:1 THF:EtOH on Mo. Samples were not rinsed in THF. (a) and (b) show the electrochemical data for two chronopotentiometry experiments where -2 mA cm^{-2} were applied for 8 minutes (a), which did not undergo working electrode drift, and 1.5 hours (b), which did undergo working electrode drift. (c) and (d) show the O^-, Cl^-, Li^- and C^- traces for the 8 minute and 1.5 hour SEI samples respectively. The 8 minute SEI sample has a relatively higher Li^- content than the 1.5 hour SEI sample. The C^- trace of the 8 minute sample is relatively stable, whereas the C^- trace for the 1.5 hour sample is increasing into the bulk of the SEI. (e) and (f) show the show mass spectra at different sputtering time intervals. It can be seen that the 1.5 hour sample has a greater quantity of heavy mass fragments, from organics, at the surface compared to the 8 minute sample. We infer that the working electrode becomes more organic over time and thus more resistive.

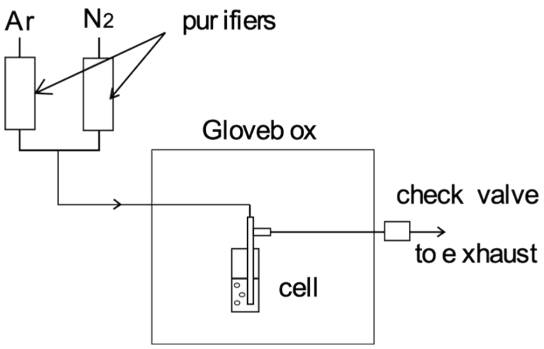

Figure S2: A diagram of the gas lines used for electrochemistry.

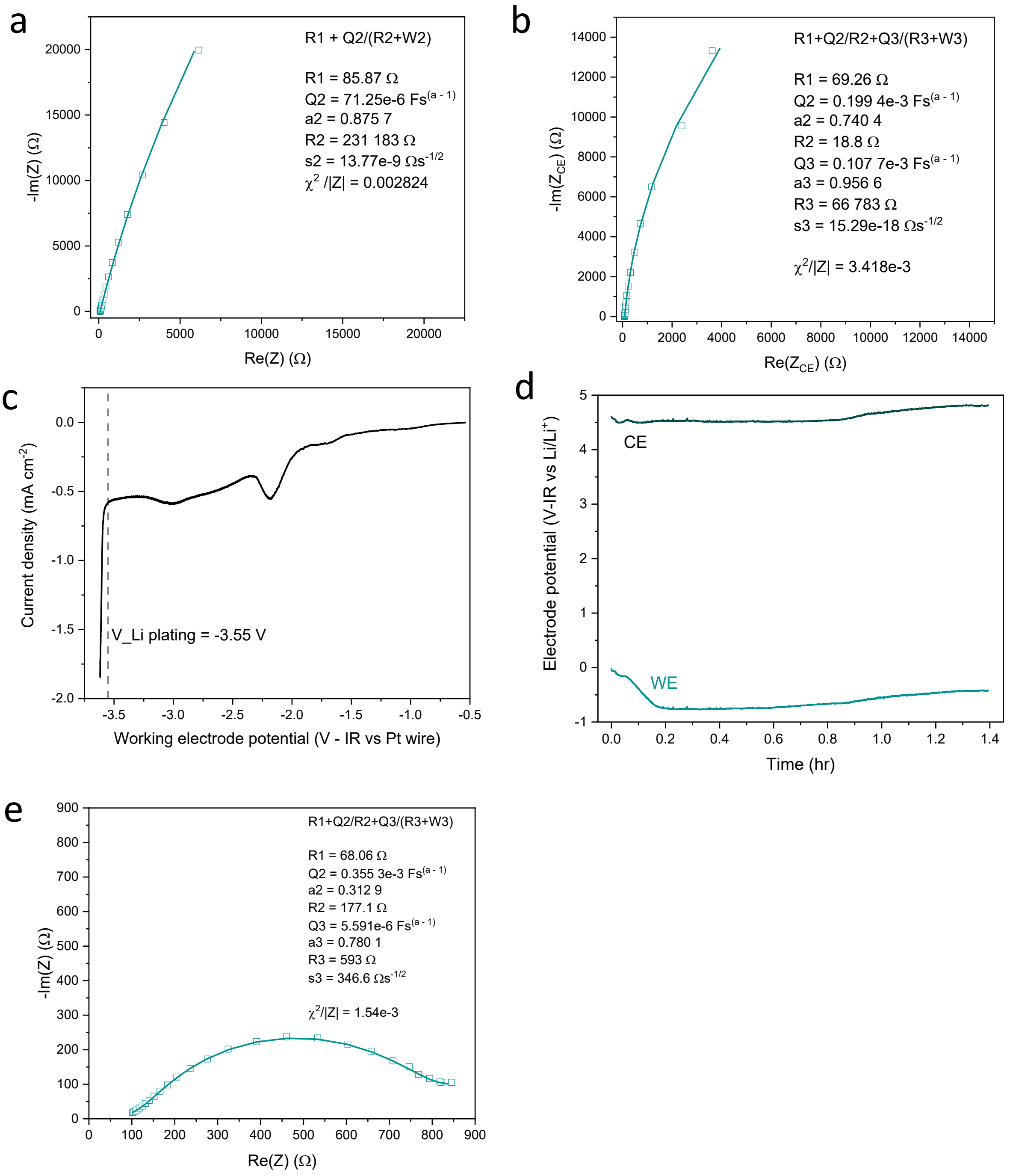

Figure S3: Examples of typical experimental protocol, shown here for a 0.6M $LiClO_4$ electrolyte on a Mo electrode. (a) Initial PEIS spectrum for the working electrode at OCV with an oscillating potential of amplitude 10 mV at frequencies between 200 kHz and 10 mHz to find the Ohmic drop for the working electrode, (b) Initial PEIS spectrum for the counter electrode at OCV with an oscillating potential of amplitude 10 mV at frequencies between 200 kHz and 10 mHz to find the Ohmic drop for the counter electrode, (c) the LSV taken to find the lithium plating potential vs the Pt wire pseudo-reference, corrected for Ohmic drop, (d) the chronopotentiometry experiment run at a constant current density of -2 mA cm^{-2} until -10C is passed corrected for ohmic drop, (e) the final PEIS spectrum taken for the working electrode to check for any significant change in ohmic drop and to determine SEI properties.

Exp ID	$LiClO_4$ Conc. (M)	Electrode	Charge passed (C)	Gas	Sample volume (µl)	Absorbance (arb. Units)	Faradaic efficiency (%)	Ohmic drop (Ω)	Initial volume (ml)	Final volume (ml)
0.2_Mo_01	0.2	Mo	-10	N_2	400	0.186 ±0.009	4.7±0.3	871	11.2	9.3
0.2_Mo_02	0.2	Mo	-10	N_2	400	0.271 ± 0.01	6.9±0.3	336	11.2	9.25
0.2_Cu	0.2	Cu	-10	N_2	400	0.302±0.03	6.7±0.7	470	11.2	8
0.2_Mo_03	0.2	Mo	-10	N_2	400	0.296 ± 0.01	6.9±0.2	774	11.2	8.4
0.6_Mo_01	0.6	Mo	-10	N_2	400	0.184± 0.007	7.4±0.3	71	11.2	9.3
0.6_Mo_02	0.6	Mo	-10	N_2	400	0.206± 0.4	7±1	69	11.2	8
0.6_Mo_03	0.6	Mo	-10	N_2	400	0.214± 0.007	8.9±0.3	79	11.2	9.6
0.6_Cu	0.6	Cu	-10	N_2	400	0.133±0.005	5.3±0.2	71	11.2	9.25
1_Mo_01	1	Mo	-10	N_2	400	0.052±0.007	3.05±0.04	50	11.2	9.3
1_Mo_02	1	Mo	-10	N_2	400	0.016±0.01	0.742±0.005	67	11.2	9.5
1_Mo_03	1	Mo	-10	N_2	400	0.179±0.016	5.99±0.07	45	11.2	8.4
1_Cu	1	Cu	-10	N_2	400	0.101±0.006	4.49±0.03	42	11.2	8.4
1.4_Mo_01	1.4	Mo	-10	N_2	400	0.026±0.035	3.6±0.4	26	11.2	9.1
1.4_Mo_02	1.4	Mo	-10	N_2	400	0.003±0.023	0±0.2	26	11.2	8
1.4_Mo_03	1.4	Mo	-10	N_2	400	0.041±0.07	5.2±0.9	22	11.2	9.5
0.6_Mo_07	0.6	Mo	-86	N_2	100	0.4 ± 0.1	6±2	54	14.2	8.8
0.6_Mo_08	0.6	Mo	-86	N_2	100	0.32 ± 0.07	5±1	60	14.2	10.35
0.6_Mo_09	0.6	Mo	-86	N_2	100	0.28 ± 0.02	3.9±0.4	71	14.2	11.5
0.2_Mo_04	0.2	Mo	-10	Ar	400	0.049 ± 0.09	Below detection limit	939	11.2	
0.2_Mo_05	0.2	Mo	0 (1.5 hr OCV)	N_2	400	0.005±0.0008	n/a	n/a	11.2	9
0.6_Mo_04	0.6	Mo	-10	Ar	400	0.001±0.002	below detection limit	137	12	9.4
0.6_Mo_05	0.6	Mo	0 (1.5 hr OCV)	N_2	400	0.0028±0.0003	n/a	n/a	11.2	8.5
0.6_Mo_10	0.6	Mo	-86	Ar	100	0.002 ± 0.002	Below detection limit	81	15	11.75
0.6_Mo_11	0.6	Mo	0 (OCV overnight)	N_2	100	0.0064± 0.0003	n/a	n/a	15	11.3

Table S1 A table of the Faradaic efficiencies obtained in this work and other key metrics.

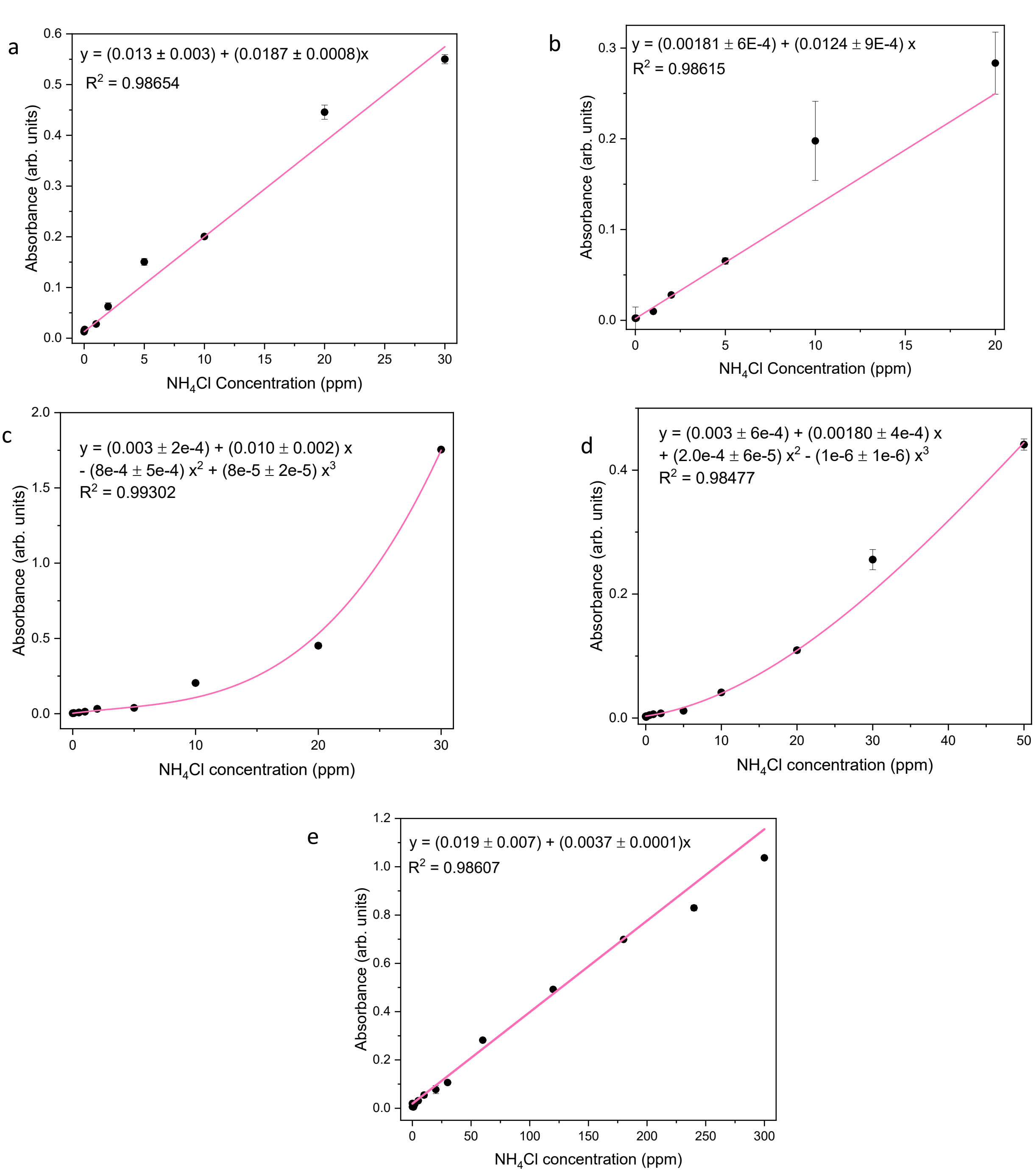

Figure S4(a – e) Calibration curves made for each of the concentrations used using 400ul electrolyte samples with added NH_4Cl : (a) 0.2 M $LiClO_4$, (b) 0.6 M $LiClO_4$, (c) 1M $LiClO_4$, (d) 1.4 M $LiClO_4$. As the salt concentration increases, the linear relationship between NH_4Cl concentration and absorbance fails. Therefore the 1M and 1.4M curves have been fitted with a cubic relationship.(e) The calibration curve used for the 0.6 M overnight experiments where 100ul electrolyte samples were used.

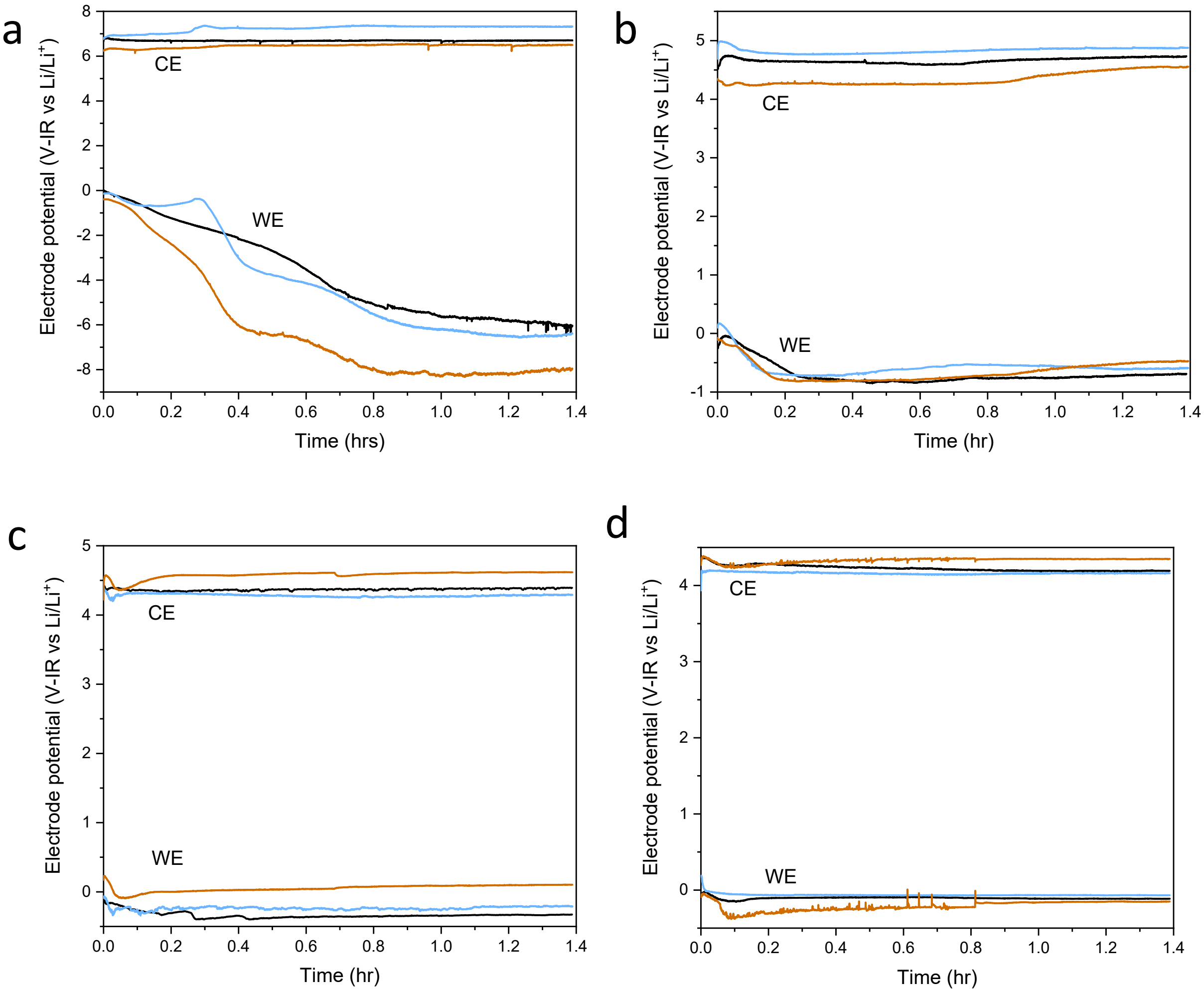

Figure S5 Chronopotentiometry data for 3 separate tests for each electrolyte paradigm, all at -2 mA cm^{-2} until -10C is passed (a) 0.2 M $LiClO_4$, (b) 0.6 M $LiClO_4$, (c) 1 M $LiClO_4$, (d) 1.4 M $LiClO_4$. Concentrations above 0.6 M $LiClO_4$ are reproducibly stable whereas 0.2 M $LiClO_4$ is reproducibly unstable.

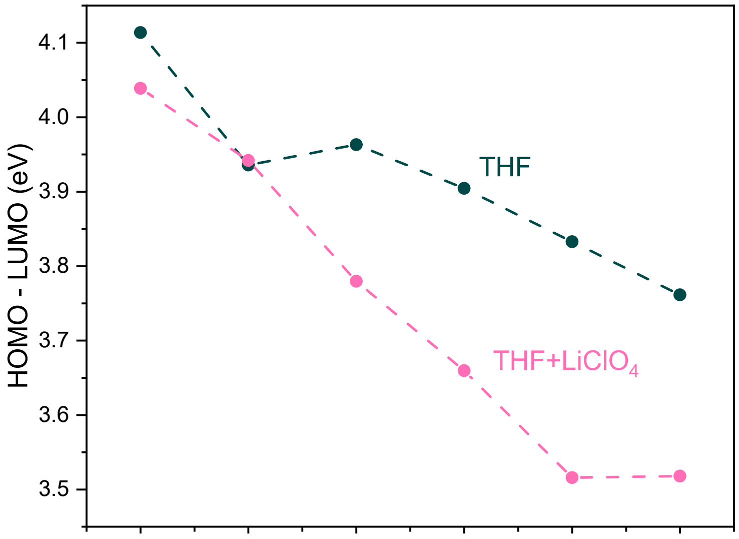

Figure S6 A plot showing the calculated HOMO-LUMO for THF with varying $LiClO_4$ content (pink) and the calculated HOMO-LUMO for the same unit cell of THF with molecules removed as if by an increasing $LiClO_4$ concentration.

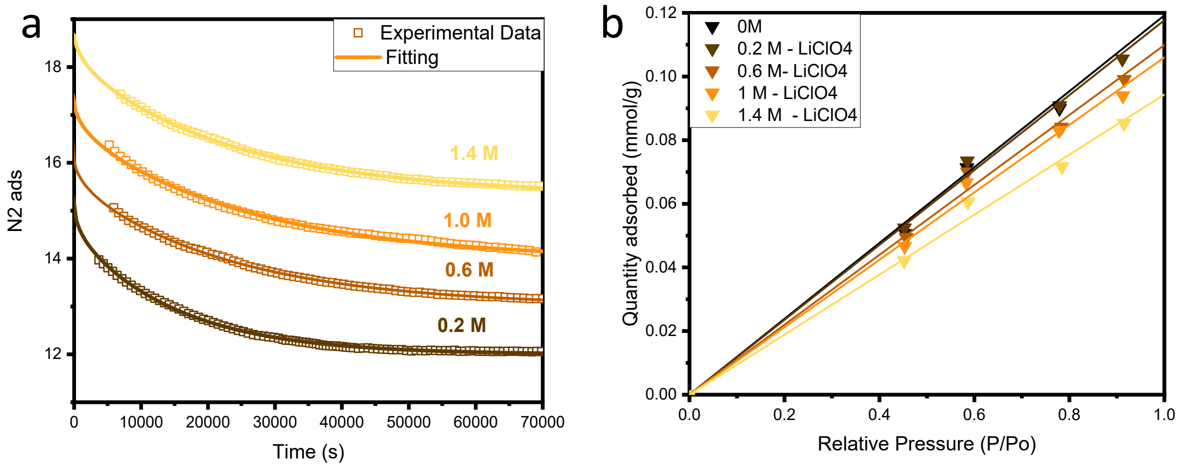

Figure S7 Calculating N_2 diffusivity and solubility (a) The fits of the solution of Fick's second law to obtain N_2 diffusivity and (b) fits to obtain N_2 solubility in 0.2 M, 0.6 M, 1 M and 1.4 M $LiClO_4$ electrolytes as described in section 8.

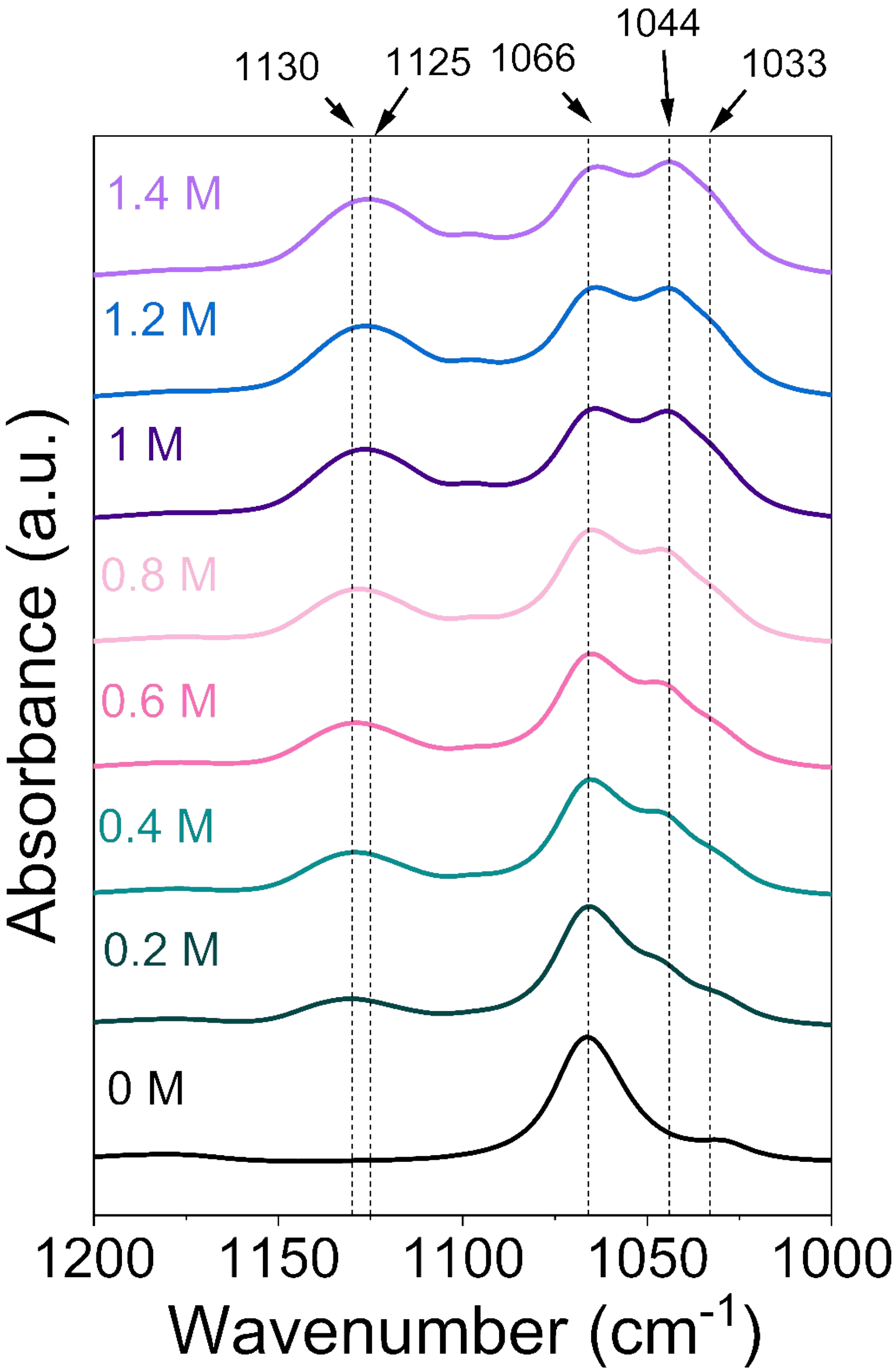

Figure S8 Fourier-Transform Infra-Red spectra of varying concentrations of $LiClO_4$ in THF. The addition of 0.2 M $LiClO_4$ to THF results in a broad peak with a maximum at 1130 cm^{-1}, which shifts its maximum to 1125 cm^{-1} with increasing concentration. Chabanel et al. assign a peak at 1130 cm^{-1} to contact ion pair formation and a peak at around 1120 cm^{-1} to dimer formation[12]. Free ClO_4^- should have a peak at 1100 cm^{-1}, but it is likely obscured by the THF peak at 1066 cm^{-1} [12]. A peak at 1044 cm^{-1} and a shoulder at 1033 cm^{-1} appear with increasing salt concentration, which are likely due to the formation of more co-ordinated Li^+ – ClO_4^- environments. Therefore it is likely that the co-ordination of lithium and perchlorate ions increases with increasing salt concentration.

Mixture	water content 1 (ppm)	water content 2 (ppm)	water content 3 (ppm)	average water content (ppm)	error (ppm)
THF + 1M undried $LiClO_4$	472.7	506.6	537	505	12
THF:EtOH 99:1 + 1M dried $LiClO_4$ (batch 1)	55.3	60.5	58.1	58	1
THF:EtOH 99:1 + 0.2M dried $LiClO_4$ (batch 1)	50.9	48.2	51	50	0.8
THF:EtOH 99:1 + 0.6M dried $LiClO_4$ (batch 1)	65	59.3		62	2
THF:EtOH 99:1 + 1.4M dried $LiClO_4$ (batch 1)	59.2	60.3	67.4	62	2
THF:EtOH 99:1 + 0.2M dried $LiClO_4$ (batch 2)	50.2	44.3		47	2
THF:EtOH 99:1 + 0.6M dried $LiClO_4$ (batch 2)	50.6	49.6	47.8	49.3	0.7
THF:EtOH 99:1 + 1M dried $LiClO_4$ (batch 2)	53.1	47.5	50	50	1
THF:EtOH 99:1 + 1.4M dried $LiClO_4$ (batch 2)	54	50.4	50.5	51	1

Table S2 Water contents of various electrolyte mixtures measured by Karl-Fischer Titration. 3 samples were taken of each mixture. Batch 1 references the first amount of salt that was dried. Batch 2 refers to the second batch of salt that was dried. There is not a significant difference in water content between the two batches or with increasing salt concentration. The undried salt contains a significant amount of water.

**Exp ID**	**R_U (Ω)**	**R_{SEI} (Ω)**	**R_{CT} (Ω)**	**Q_{SEI} (Fs^{a-1})**	**a_{SEI}**	**Q_{DL} (Fs^{a-1})**	**a_{DL}**	**W (Ω $s^{-1/2}$)**	**Fit**
0.2_Cu[a]	314.1	2861	1988	33.71×10^{-6}	0.3716	2.812×10^{-6}	0.8504	5.176×10^{-21}	0.0222
0.6_Cu[a]	71.14	361.1	545.7	11.83×10^{-6}	0.7568	0.368×10^{-3}	0.484	0.130×10^{-9}	0.007674
1_Cu[a]	42.4	167.8	24.14	10.55×10^{-6}	0.8072	0.108×10^{-3}	0.5423	122	0.005273
0.2_Mo_03	774	5789	2343	5.762×10^{-6}	0.4921	1.668×10^{-6}	0.8104	0.415×10^{-21}	0.04429
0.2_Mo_02	336	3537	2561	8.415×10^{-6}	0.5508	0.747×10^{-6}	1	7.408×10^{-15}	0.05787
0.2_Mo_01[b]	350	6596	2017	0.139×10^{-3}	0.1925	5.981×10^{-6}	0.5597	0.2×10^{-24}	0.0106
0.6_Mo_03	79	603	724	7.925×10^{-6}	0.7361	0.125×10^{-3}	0.1986	597	0.01323
0.6_Mo_02	68	177	593	0.3553×10^{-3}	0.312	5.591×10^{-6}	0.7801	347	0.00154
0.6_Mo_01	63	880	1221	0.132×10^{-3}	0.4427	4.846×10^{-6}	0.8244	243	0.0304
1_Mo_02	68	188	38	7.153×10^{-6}	0.8503	61.13×10^{-6}	0.6681	116	0.00105
1_Mo_03	44	112	175	12.17×10^{-6}	0.8091	42.11×10^{-6}	0.6984	66	0.00541
1_Mo_01	51	22	66	8.163×10^{-6}	0.8481	6.482×10^{-6}	0.9121	0.9959	0.05612

Table S3 Fitting parameters for impedance spectra shown in figure 5 (demarked [a]) and for some other experiments reported in this work for spectra taken after -10C is passed during chronopotentiometry. [b] Especially noisy data, fit only on reduced number of points

Name	Li	ClO4	THF	Total atoms
0.5 M LiClO4	1	1	29	383
1.0 M LiClO4	2	2	27	363
1.5 M LiClO4	3	3	26	356
2.0 M LiClO4	4	4	25	349
2.5 M LiClO4	5	5	23	329
3.0 M LiClO4	6	6	22	322

Table S4: Showing the ion, THF and total number of atoms in the simulated AIMD electrolytes.

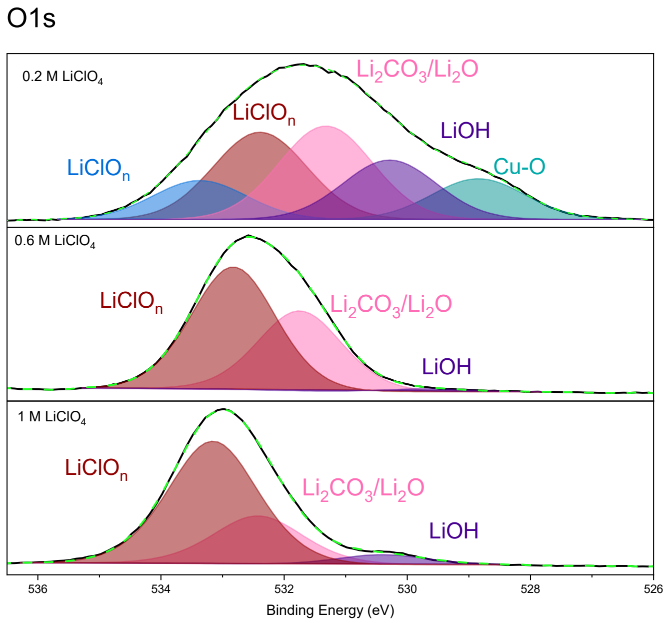

Figure S9 The O1s core levels for the 0.2 M, 0.6 M, and 1 M $LiClO_4$ samples on Cu. There are no clear features; an attempt at fitting has been made, but no real conclusions can be drawn.

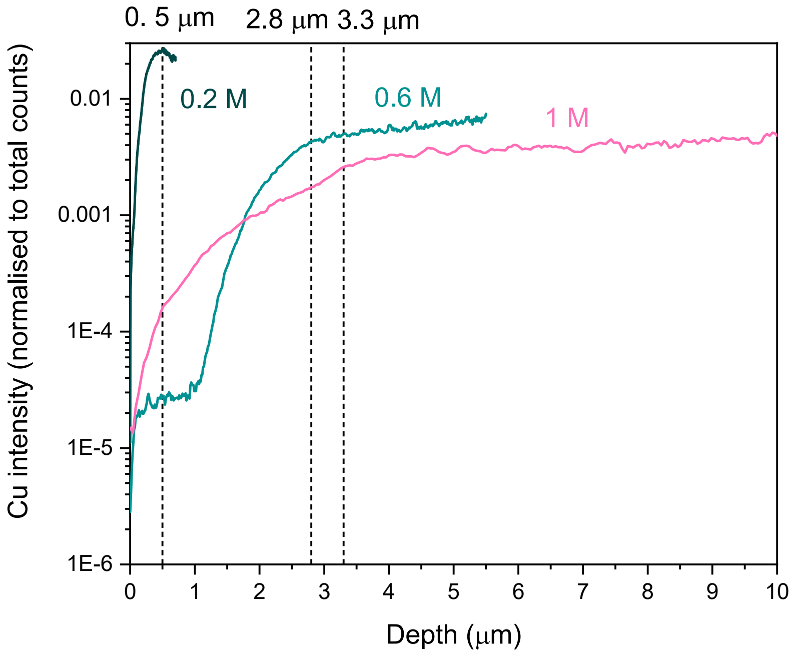

Figure S10 Cu intensity normalised to total counts vs depth of sputtering, calculated using the total crater depth and sputter time. The end of the SEI is defined as the turning point of the Cu signal. Crater depths were 0.7 μm, 5.5 μm and 15 μm for the 0.2M, 0.6M and 1M SEI samples respectively. Although Cu_2^- could have been used to determine the Cu surface to eliminate the possibility of a thick CuO layer, the Cu^- signal had a much higher intensity so was deemed a more reliable measure of approximate SEI thickness. It is also likely that the CuO would be reduced under the reducing conditions used in this experiment.

References:

1. Mei H, Laws TS, Terlier T, et al. Characterization of polymeric surfaces and interfaces using <scp>time-of-flight</scp> secondary ion mass spectrometry. *Journal of Polymer Science* 2022; 60: 1174–1198.

2. K. Hirokawa, Z. Li, A. Tanaka. Role of electronegativity in the qualitative inference of the TOF–SIMS fragment pattern of inorganic compounds. *Fresenius J Anal Chem* ; 370.

3. Li Z, Hirokawa K. Ga + primary ion ToF-SIMS fragment pattern of inorganic compounds and metals. *Appl Surf Sci* 2003; 220: 136–153.

4. Lazouski N, Schiffer ZJ, Williams K, et al. Understanding Continuous Lithium-Mediated Electrochemical Nitrogen Reduction SI. *Joule* 2019; 3: 1127–1139.

5. Giner-Sanz JJ, Leverick GM, Giordano L, et al. Alkali Metal Salt Interference on the Salicylate Method for Quantifying Ammonia from Nitrogen Reduction. *ECS Advances* 2022; 1: 024501.

6. Andersen SZ, Čolić V, Yang S, et al. A rigorous electrochemical ammonia synthesis protocol with quantitative isotope measurements. *Nature* 2019; 570: 504–508.

7. Zubeir LF, Lipman PJL, van der Schaaf J, et al. Novel method for determination of gas solubilities in low vapor pressure liquids. *AIChE Journal* 2017; 63: 2981–2986.

8. Hjorth Larsen A, JØrgen Mortensen J, Blomqvist J, et al. The atomic simulation environment - A Python library for working with atoms. *Journal of Physics Condensed Matter*; 29. Epub ahead of print 7 June 2017. DOI: 10.1088/1361-648X/aa680e.

9. Enkovaara J, Rostgaard C, Mortensen JJ, et al. Electronic structure calculations with GPAW: A real-space implementation of the projector augmented-wave method. *Journal of Physics Condensed Matter*; 22. Epub ahead of print 2010. DOI: 10.1088/0953-8984/22/25/253202.

10. Berendsen HJC, Postma JPM, van Gunsteren WF, et al. Molecular dynamics with coupling to an external bath. *J Chem Phys* 1984; 81: 3684–3690.

11. Walter M, Moseler M. Ab Initio Wavelength-Dependent Raman Spectra: Placzek Approximation and beyond. *J Chem Theory Comput* 2020; 16: 576–586.

12. Chabanel M, Legoff D, Touaj K. Aggregation of perchlorates in aprotic donor solvents. Part 1.—Lithium and sodium perchlorates. *J Chem Soc, Faraday Trans* 1996; 92: 4199–4205.
